# Computational modelling of solvent effects in a prolific solvatomorphic porous organic cage†
†Electronic supplementary information (ESI) available. CCDC 1575910–1575914. For ESI and crystallographic data in CIF or other electronic format see DOI: 10.1039/c8fd00031j


**DOI:** 10.1039/c8fd00031j

**Published:** 2018-04-16

**Authors:** David P. McMahon, Andrew Stephenson, Samantha Y. Chong, Marc A. Little, James T. A. Jones, Andrew I. Cooper, Graeme M. Day

**Affiliations:** a Computational Systems Chemistry , School of Chemistry , University of Southampton , SO17 1BJ , UK . Email: g.m.day@soton.ac.uk; b Department of Chemistry and Materials Innovation Factory , University of Liverpool , Crown St. , Liverpool L69 7ZD , UK . Email: aicooper@liverpool.ac.uk

## Abstract

Crystal structure prediction methods can enable the *in silico* design of functional molecular crystals, but solvent effects can have a major influence on relative lattice energies, sometimes thwarting predictions. This is particularly true for porous solids, where solvent included in the pores can have an important energetic contribution. We present a Monte Carlo solvent insertion procedure for predicting the solvent filling of porous structures from crystal structure prediction landscapes, tested using a highly solvatomorphic porous organic cage molecule, **CC1**. Using this method, we can understand why the predicted global energy minimum structure for **CC1** is never observed from solvent crystallisation. We also explain the formation of three different solvatomorphs of **CC1** from three structurally-similar chlorinated solvents. Calculated solvent stabilisation energies are found to correlate with experimental results from thermogravimetric analysis, suggesting a future computational framework for *a priori* materials design that factors in solvation effects.

## Abstract 2

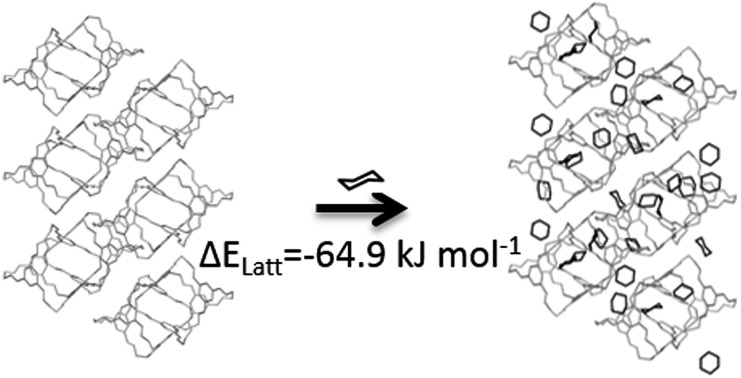
A computational approach has been developed to assess the effect of solvent stabilisation on the predicted crystal structures of a porous organic cage.

## Introduction

A fundamental goal for computational chemistry is the *in silico* design of functional materials. Computational methods can reduce our reliance on trial and error experiments, saving laboratory time. Because materials properties are defined by structure, *in silico* methods for screening hypothetical functional molecules must involve a structural hypothesis; this can be done using analogy, empirical rules, or *ab initio* solid-state structure prediction. For molecular crystals, empirical rules are unreliable because the crystal packing is determined by the summation of many weak interactions, rather than a single, dominant interaction. A small change in molecular structure often alters the crystal structure completely. This means that intuitive design strategies for molecular crystals will frequently fail or, at least, fail to capture the true complexity of the potential crystallisation landscape for a given molecule. Computational methods developed for crystal structure prediction (CSP) give us the potential to predict structure from the molecular building blocks alone.^1^,^2^ So far, CSP methods have largely focused on pharmaceutical molecules,^3^–^5^ but they have also been applied in areas of materials chemistry, such as organic semiconductors^6^–^9^ and porous organic molecular crystals.^10^–^13^

An important factor that is usually overlooked in CSP is the effect of solvent on the relative crystal structure stabilities and, hence, the structure that is predicted to form. Alternative crystal structures are often predicted to be close in lattice energy and the interaction of solvent molecules during crystallisation can affect the stabilisation of one potential packing arrangement with respect to another. Frequently, the influence of solvent is limited to interactions with the surface of a crystal. The total free energy of a crystal has contributions from the bulk and surface energies, such that structures with the lowest surface energies can be more stable than those with the lowest bulk energy. The balance between bulk and surface energies depends on crystallite size and shape, with surface terms increasing for small crystallite sizes.^14^ While the bulk energy of a crystal is unaffected by the solvent environment, surface energies can be strongly influenced. Thus, different solvents can stabilise different structures and solvent stabilisation of crystal surfaces can have an important influence at the critical nucleation stage, leading to the growth of metastable phases. These effects on polymorph stabilities have been modelled for known polymorphs of inorganic^15^,^16^ and organic^17^ materials using explicit^15^,^16^ or continuum^17^ models of solvent on the dominant crystal faces.

The influence of solvent becomes especially important for porous crystals, which tend to crystallise with solvent in the pore channels. Solvated polymorphs, or “solvatomorphs”, are of intrinsic interest because solvatomorphs can have different physical properties.^18^–^21^ For porous crystals, different porous polymorphs can be isolated by desolvating solvatomorphs with different crystal structures.^10^,^22^,^23^ This can be used to an advantage: for example, we showed previously that 1,4-dioxane acts as a directing solvent to induce the formation of a specific highly-porous crystal packing of organic cage molecules.^23^ The solvent molecules in a porous molecular crystal can also be an integral part of the crystal structure, and hence the removal of solvent from solvatomorphs with retention of the crystal packing of the host structure is not always possible.^24^,^25^

It has been shown that the inclusion frameworks of organic molecules often correspond to local minima on the lattice energy surface, even in the absence of the guest solvent molecules.^26^ They can therefore be located using CSP methods, usually at energies significantly above the global lattice energy minimum. These structures can be identified because they contain voids of the right size to accommodate molecular guests. It is not usually clear, *a priori*, which solvent will stabilise which particular crystal packing.^27^ As such, predicting the influence of solvent on relative crystal lattice energies is of strong value because it could allow crystallisation conditions to be explored *in silico* to target specific polymorphs. For example, we recently used solvent stabilisation calculations to explain why a hydrogen bonded molecule, triptycene-tris(benzimidazolone), crystallises as a series of porous polymorphs and not as a dense, non-porous phase, which is the predicted global lattice energy minimum structure.^10^

**Fig. 1:**
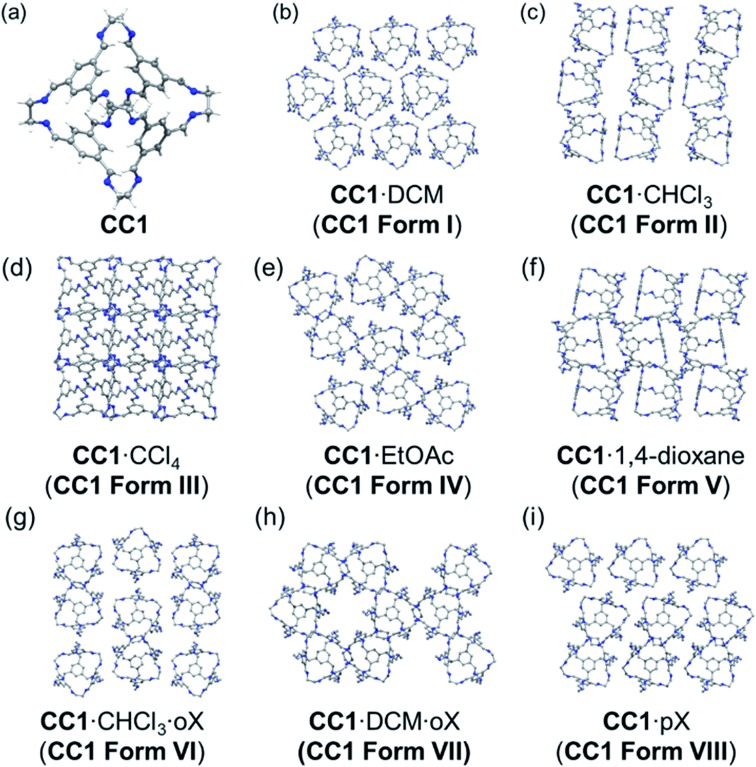
Experimentally determined crystal structures of the solvatomorphs of **CC1** (a) shown as a 3 × 3 grid of cages from each structure (b–i). Solvent molecules omitted for clarity. *o*X = *ortho*-xylene and *p*X = *para*-xylene.

Here, we investigate the effect of solvent on the crystallisation of an ethylenediamine-derived [4 + 6] imine cage, **CC1** (Fig. 1a).^22^,^28^ **CC1** is a good candidate for studying solvent effects on polymorphism: unlike many imine cages, where bulky vertices control the crystal packing, **CC1** is nearly spherical and it has many structures of similar energy on its predicted crystal energy landscape.^12^ The **CC1**-α′ polymorph is formed by desolvation of an ethyl acetate (EtOAc) solvate, **CC1**·2.5(EtOAc), while **CC1**-β′ is formed by desolvation of a dichloromethane (DCM) solvate, **CC1**·2.5(DCM).^22^ The desolvated crystal structures are related directly to their respective solvatomorphs and the two different polymorphs have different properties: **CC1**-β′ is porous to nitrogen while **CC1**-α′ is not.^22^ The phase behaviour of **CC1** is not limited to these polymorphs: for example, recrystallisation from DCM/mesitylene affords **CC1**·4(mesitylene) (*P*1[combining macron]),^29^ and recrystallisation from DCM/1,4-dioxane affords 2(**CC1**)·7(1,4-dioxane) (*P*2_1_/*c*).^23^ Hence, the solid form landscape of **CC1** is particularly rich and non-intuitive from an experimental perspective.

We present here the preparation and characterisation of five new solvatomorphs of **CC1** (Fig. 1), which exist as either single solvates (one cocrystallised solvent) or as bisolvates (two different cocrystallised solvents). By using a Monte Carlo solvent filling procedure in conjunction with lattice energy minimisation, we show that all the solvatomorphs are solvent stabilised compared to the hypothetical global minimum on the CSP landscape. In addition, we show for three different chlorinated solvents that the experimentally obtained crystal solvatomorph is correctly predicted by solvent stabilisation calculations; that is, the observed solvatomorph is predicted to be more stabilised by the solvent used for its crystallisation than any of the alternative **CC1** structures that were tested. This is further corroborated by thermogravimetric analysis (TGA) of the experimental solvates, which shows a correlation between the experimental stability of the solvatomorph and the computed solvent stabilisation energy.

## Methods

### Crystallisation and characterisation

Crystals of **CC1** were obtained using either slow evaporation, vapour diffusion, or layering methods, details of which are provided in the ESI.† **CC1** is soluble in chlorinated solvents such as DCM and CHCl_3_ but poorly soluble in non-polar solvents, such as xylenes, and also in some polar solvents, such as EtOAc. Hence, combinations of solvents were used in some cases for the crystallisation of **CC1** where limited solubility did not allow a single solvent to be used.

Single crystal X-ray data sets were measured on a Rigaku MicroMax-007 HF rotating anode diffractometer (Mo-Kα radiation, *λ* = 0.71073 Å, Kappa 4-circle goniometer, Saturn 724+ detector); at beamline I19, Diamond Light Source, UK using silicon double crystal monochromated synchrotron radiation (*λ* = 0.6889 Å, Saturn 724+ detector);^30^ or at beamline 11.3.1, Advanced Light Source, Berkeley, USA, using silicon monochromated synchrotron radiation (*λ* = 0.7749 Å, APEX-II detector). Solvated single crystals were immersed in a protective oil, mounted on a MiTeGen loop, and flash cooled under a dry N_2_ gas flow. Absorption corrections, using the multi-scan method, were performed with the program SADABS.^31^ Structures were solved with SHELXD,^32^ or by direct methods using SHELXS,^33^ and refined by full-matrix least squares on |*F*|^2^ by SHELXL.^34^ Supplementary CIFs, that include structure factors, are available free of charge from the Cambridge Crystallographic Data Centre (CCDC). 2(**CC1**)·7.75(DCM) #; 1575913, 2(**CC1**)·11.32(CHCl_3_) #; 1575914, 2(**CC1**)·10(CCl_4_) #; 1575912, CC1·2(*o*X)·CHCl_3_ #; 1575911, and CC1·*o*X·DCM #; 1575910. For full refinement details, see the ESI (Tables S1 and S2, Fig. S1–S7†).

Powder X-ray diffraction data was collected in transmission mode using the Mythen-II position sensitive detector and capillary spinner on the I11 beamline at the Diamond Light Source (*λ* = 0.827157 Å). The sample was contained in a 0.5 mm diameter borosilicate glass capillary. The structure of CC1·1.47(*p*X) was solved using the simulated annealing routine implemented in Topas-Academic.^35^ For PXRD refinement details, see the ESI (Fig. S8 and S9†).

TGA was carried out using a TA Q5000IR analyser with an automated vertical overhead thermobalance. Samples were heated at a rate of 10 °C min^–1^. *T*_onset_ was calculated using the in-built feature in the software ‘TA Universal Analysis’. TGA curves and calculated *T*_onset_ values are in the ESI.†

### Crystal structure prediction

As a reference crystal energy landscape for **CC1** (Fig. 3), we use the structures from Case *et al.*,^36^ which was generated by quasi-random sampling with one molecule in the asymmetric unit (*Z*′ = 1) in 16 common space groups (*P*1, *P*2_1_, *C*2, *P*2_1_2_1_2_1_, *P*2_1_2_1_2, *C*222_1_, *P*4_1_2_1_2, *R*3, *P*1[combining macron], *Cc*, *P*2_1_/*c*, *C*2/*c*, *Pna*2_1_, *Pbcn*, *Pbca*, and *Pnma*). This search used 5000 accepted trial crystal structures in each space group, in which molecular clashes were relieved using the SAT-expand method described in ref. 36. This previously published set of predicted crystal structures for **CC1** was supplemented by additional crystal structure searching where we identified that the sampling in our previous set was not complete. Specifically, we added structures in *C*2/*c* with *Z*′ = 1 and sampled those space groups with two independent molecules (*Z*′ = 2) where this symmetry is observed in one of the known **CC1** structures. These additional crystal structure searches were performed using the Global Lattice Energy Explorer (GLEE) software,^36^ which performs a quasi-random sampling of lattice dimensions, molecular positions and orientations. The quasi-random sampling used to generate the CSP landscape has the advantage over other global optimisation strategies that the sampling of higher energy regions of the landscape is given equal importance to sampling the low energy region near the global minimum. We find (as discussed below) that the **CC1** frameworks of observed solvates can be high in energy and so could be easily missed by more aggressive global optimisation algorithms.

Lattice energy minimisation calculations used the DMACRYS^37^ software in which molecular geometries are held rigid at the gas phase B3LYP/6-31G** geometry. Intermolecular interactions are described using an empirically parameterised atom–atom repulsion–dispersion model (W99 (ref. 38)) and a distributed multipole electrostatic model, using a distributed multipole analysis^39^ (DMA) of the B3LYP/6-31G** electron density with multipoles up to hexadecapole on each atom. Ewald summation was used to calculate the charge–charge, charge–dipole, and dipole–dipole interactions; all other intermolecular interactions were subject to a 30 Å cutoff. Full details are provided in the ESI.†

### Solvent insertion calculations

**Fig. 2:**
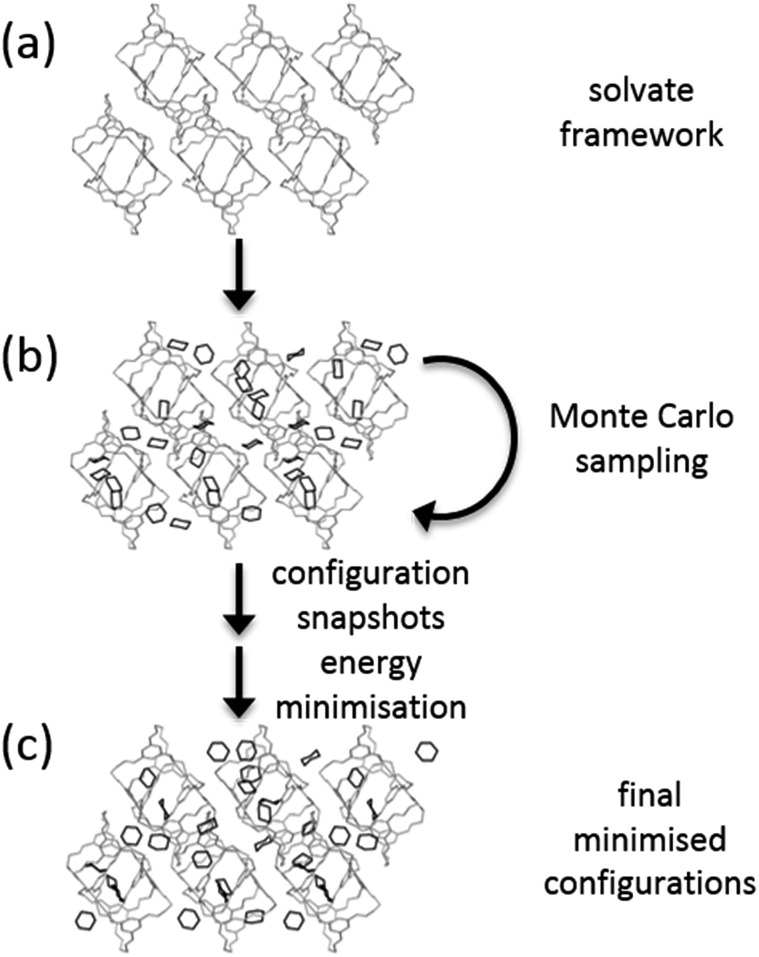
Scheme of the Monte Carlo solvent loading calculations. Starting from a **CC1** host generated by crystal structure prediction (a), Monte Carlo steps are used to sample configurations of solvent molecules (b), and snapshots from the Monte Carlo sampling are subjected to lattice energy minimisation (c).

A Monte Carlo (MC) approach was used to insert solvent molecules into **CC1** host frameworks (Fig. 2). MC calculations were performed using Towhee-7.10 (ref. 40) on the CSP structures corresponding to all of the observed forms of **CC1**, as well as the global lattice energy minimum structure determined from CSP. 10 000 cycle MC simulations were run in the NVT ensemble using a combination of translational, rotational and configuration-bias regrowth and reinsertion moves (the distribution of moves is given in the ESI†). For bisolvates, we also included two-molecule centre-of-mass switch moves. Simulations were run for a range of solvent loading ratios (**CC1** : solvent = 1 : *N*, *N* = 1,2,3…) until no further solvent could be added without disrupting the **CC1** packing.

We attempted insertion of each solvent (CHCl_3_, DCM, CCl_4_, EtOAc, 1,4-dioxane, *p*-xylene) and solvent mixtures (CHCl_3_ : *o*-xylene and DCM : *o*-xylene) into the **CC1** framework that is observed to form with that solvent. For mixed solvent systems (bisolvates), the experimental solvent ratios were used during the solvent insertion calculations. It is a future challenge to extend our current methods to assess mixed solvent systems without experimental information on the solvent ratio in the solvated crystal structure. We also used the Monte Carlo approach to load five of the single solvents (CHCl_3_, DCM, CCl_4_, EtOAc, 1,4-dioxane) into all of the solvent frameworks and the CSP global minimum crystal structure.

An artificially high temperature of *T* = 5000 K was used to ensure good sampling of solvent configurations within the crystal structures. Frames sampled at 10 cycle intervals were lattice energy minimised with the same energy model used in the CSP calculations (W99 + DMA). No space group symmetry was enforced during solvent sampling or lattice energy minimisation. Solvated structures that led to large distortions of the **CC1** framework were discarded. This was tested by comparison of the **CC1** arrangement within the solvated structure to the original CSP structure using the COMPACK algorithm.^41^

The lattice energies of the solvated crystal structures were then corrected for the cost of removing *N* molecules of solvent from the pure phase:1
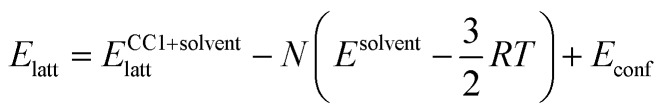
where *E*^solvent^ was calculated from NVT MC simulations on pure solvent at 300 K, in which the solvent densities were fixed at the experimental density.

The 
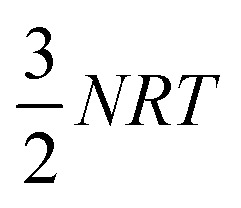
 correction accounts for the change in the kinetic energy contribution of the solvent molecules to the internal energy. We assume solvent molecule rotational motion to be free in the bulk solvent phase, and to become librational vibrations in the solvate crystals and use equipartition estimates of the energy. *E*_conf_ is an energy correction between the conformations of **CC1**, which is added to any structure with a higher energy conformation.

## Results and discussion

### Crystallisation

The various experimental solvatomorphs of **CC1** are shown in Fig. 1. **CC1** crystallises from CHCl_3_ as 2(**CC1**)·11.32(CHCl_3_) in the triclinic space group, *P*1[combining macron]. In this structure, ordered CHCl_3_ molecules were found in the cage cavities (Fig. S1†). In addition, a mixture of ordered and disordered CHCl_3_ molecules was found in extrinsic 1-D channels running along the *c*-axis. By contrast, **CC1** crystallises from a CCl_4_/CHCl_3_ mixture as 2(**CC1**)·10(CCl_4_) in the cubic space group, *F*23. In this structure, ordered CCl_4_ molecules were found in the cage cavities, with the Cl atoms pointing toward the four tetrahedrally-arranged cage windows (Fig. S2 and S3†). 2(**CC1**)·10(CCl_4_) was formed by layering CCl_4_ onto a solution of **CC1** dissolved in CHCl_3_, yet the refined crystal structure indicates that only CCl_4_ crystallised with **CC1**, and not CHCl_3_, which was also confirmed by ^1^H NMR (Fig. S4†). In the published crystal structure of **CC1**·2.5(DCM) (*R*3), the dichloromethane (DCM) molecules were poorly resolved.^22^ By repeating this crystallisation, we were able to isolate better quality crystals and the new structure was refined as 2(**CC1**)·7.75(DCM). There was still some disorder evident in 2(**CC1**)·7.75(DCM), but it was possible to accurately determine solvent positions and occupancies (Fig. S5†). Interestingly, the crystal structures obtained from these three chlorinated solvents (CHCl_3_, CCl_4_, and DCM) are all quite different (Fig. 1b–d), despite the relatively weak interactions between the solvents and the cage molecules.

In **CC1**·2.5(EtOAc) (*C*2/*c*), one EtOAc molecule was located in the **CC1** cavity with additional disordered EtOAc molecules being found throughout the narrow 1-D channel along the *b*-axis.^28^ In 2(**CC1**)·7(1,4-dioxane) (*P*2_1_/*c*), 1,4-dioxane sits in the window of two adjacent cage molecules and directs **CC1** to pack window-to-window. Extrinsic pockets also exist between **CC1** molecules filled with two 1,4-dioxane molecules.^23^,^28^

By using anti-solvent vapour diffusion crystallisations with *ortho*-xylene (*o*X) and *para*-xylene (*p*X), we isolated three additional solvatomorphs with different **CC1** crystal structures: **CC1**·DCM·*o*X (*P*2_1_2_1_2_1_); **CC1**·CHCl_3_·2(*o*X) (*P*2_1_/*n*); and **CC1**·1.47(*p*X) (*P*2_1_) (Fig. 1). **CC1**·CHCl_3_·2(*o*X) was obtained from a mixture of chloroform and *o*X, crystallising in the form of thin needles (Fig. S6†). In this structure, CHCl_3_ occupies the cage centres and *o*X occupies the interstitial spaces between the cage molecules to form extended, solvent-filled 1-D channels along the crystallographic *a*-axis. When DCM and *o*X were used, **CC1**·DCM·*o*X was obtained (Fig. S7†). As with the CHCl_3_ analogue, the chlorinated solvent is in the cage centre. In this case, *o*X is in 1-D channels along the crystallographic *b*-axis and the resultant packing motif of **CC1** is very different. In **CC1**·DCM·*o*X, the channels are formed by arrangement of 6 **CC1** molecules such that the *o*X sits in a relatively large hexagonal-shaped channel (Fig. 1h). By contrast, in **CC1**·CHCl_3_·*o*X, the *o*X molecules sit in smaller channels surrounded by just 4 cages (Fig. 1g). This leads to a different **CC1** : solvent ratio; in both cases, there is one chlorinated solvent molecule per cage, but in the CHCl_3_·*o*X solvate, there are two *o*X molecules per cage, while in the DCM·*o*X solvate there is only one. We were unable to obtain suitable single crystals for the *p*X solvate of **CC1**, **CC1**·1.47(*p*X), but its structure was solved by powder X-ray diffraction (PXRD) in the monoclinic space group, *P*2_1_ (Fig. S8 and S9†). There are no solvent-filled channels between cages in this solvatomorph (Fig. 1i); instead, the *p*X solvent sits inside the cage cavity and also in the interstitial voids, pointing into the cage window. The intrinsic site is partially occupied, with an occupancy of less than 50% in contrast to the extrinsic window site, which shows full occupancy. The total number of *p*X guests per cage is 1.471(2).

### Crystal structure prediction

**Fig. 3:**
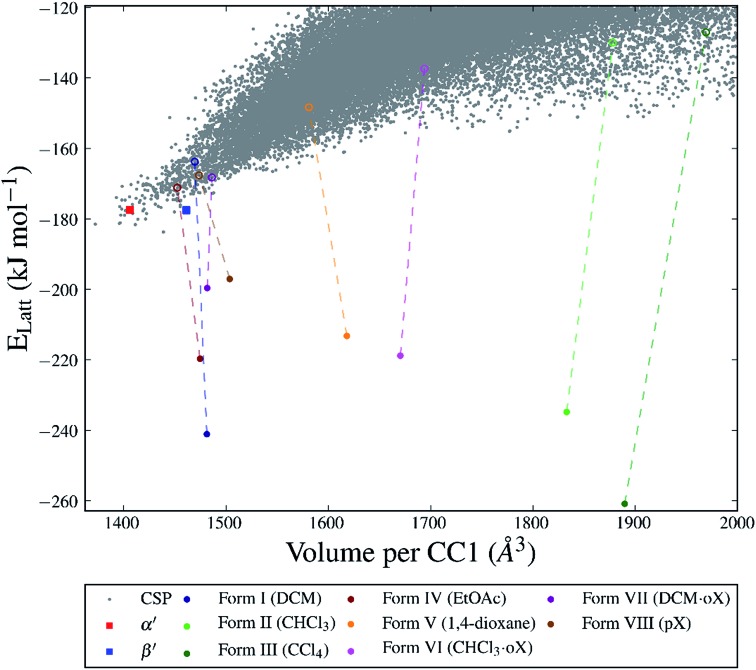
CSP energy landscape for **CC1** (grey points) also showing the calculated lattice energies of the desolvated structures (**Forms I–VIII**, unfilled circles) and after solvent stabilisation (filled circles). Each solvent-filled structure is represented by the lowest energy configuration from the Monte Carlo sampling. The experimentally determined α′ and β′ polymorphs are shown as squares.

For many of the chiral organic cages that we have studied previously, we predicted a large energy gap between the global lattice energy minimum and the bulk of the predicted structures;^12^,^13^,^42^ for example, this energy gap is 26 kJ mol^–1^ for **CC3**-R. In comparison, for this structurally related cage, **CC1**, we predicted a lattice energy surface with no large energy gaps between structures (Fig. 3). Less dense structures on the **CC1** landscape exhibit, in general, higher relative lattice energies, reflecting the energetic cost of void space in the crystal structure. The shapes and sizes of the extrinsic voids in each of the predicted structures are different, but it is non-trivial to determine what effect different crystallisation solvents will have on the energies of these structures. Therefore, it is not possible to simply determine which crystal packing will be formed from a given crystallisation solvent system using the CSP results for **CC1** alone.

The two unsolvated experimental polymorphs, **CC1**-α′ and **CC1**-β′, are both present in the predicted set of structures, 6.3 and 6.2 kJ mol^–1^ above the global minimum in the energy landscape, respectively, which is within the usual energetic range of polymorphism^43^ in organic molecular crystals. For comparison, we calculated lattice energies of the various experimental solvates (Fig. 1) after artificial removal of the lattice solvent and subsequent lattice energy minimisation. All of these ‘artificial desolvates’ are located on the CSP landscape. The resulting structures are plotted on the CSP crystal energy landscape (Fig. 3, unfilled coloured circles). These results confirm that all 7 of the experimental solvate packings for **CC1** can be located using CSP methods without including solvent molecules in the calculation. In the case of the high-energy **Form II** structure, the **CC1** framework was only found after additional sampling in the observed space group (*C*2/*c*, *Z*′ = 1); this is a particularly high energy structure (53.8 kJ mol^–1^ above the global minimum) and there are many local minima at such high energies, making the location of all local minima very challenging.

The significant challenge, then, is to identify which of the many predicted structures will be stabilised by which solvent or solvent mixture. The first step, tackled here, is to understand the relevant energetics: that is, the energy range on the CSP landscape where solvates are found and the energetic stabilisation that arises from the inclusion of solvent molecules. The calculated lattice energies for the artificially desolvated **CC1** structures range from 12.6 to 56.7 kJ mol^–1^ above the global lattice energy minimum – that is, the lowest energy solvent-free crystal packing predicted for **CC1** (Fig. 3). Some of these solvate frameworks are therefore much higher in energy than typical organic polymorphs; hence, these packing arrangements of **CC1** are only observed because of the energetic influence of solvent templating.

### Solvent stabilisation for observed solvatomorphs

We first investigated the solvent stabilisation energies for the experimentally-observed solvatomorphs by inserting the relevant solvent into the respective solvent-free structure on the CSP energy landscape. To do this, a Monte Carlo procedure was used to insert solvent into the CSP predicted matches to each known **CC1** solvate structure at a range of solvent : **CC1** loadings.

**Fig. 4:**
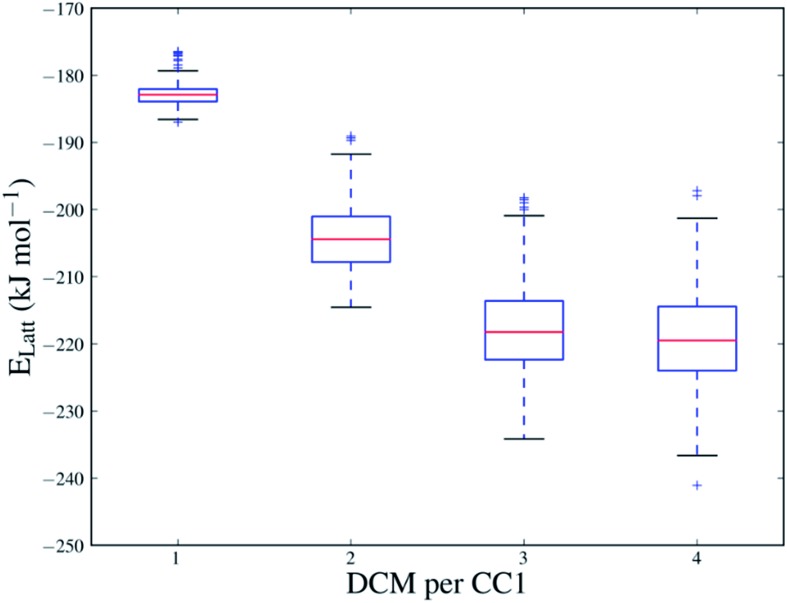
Box plot of the energies of solvated structures of DCM in **CC1 Form I** at a range of solvent loadings (**CC1** : DCM = 1 : *N*, *N* = 1–4). Red lines indicate the median energy across the set of configurations for each value of *N*. The upper and lower limits of the box indicate the 1^st^ and 3^rd^ quartiles. The whiskers show the range of the calculated energies within 1.5× the interquartile range of the box limits and outliers are denoted by a cross.

The sampling performed in the Monte Carlo approach is important in evaluating the solvent stabilisation energies because of the strong dependence of the energy on the positions and orientations of the solvent molecules in the **CC1** framework. Fig. 4 shows the range of energies for the sampled configurations of DCM in **Form I** at all sampled solvent loadings (all sampled configurations of each solvent system are also shown in Fig. S11†). At *N* = 4, where the **CC1** framework is fully solvated, the different solvent configurations produced by the Monte Carlo sampling cover a range of approximately 40 kJ mol^–1^ and only a few of these are close in energy to the lowest energy configuration.

To evaluate the energetic stabilisation, we assume ordered solvent in the voids of the **CC1** structures and, so, we take the most stable calculated solvent arrangement in each of the host **CC1** frameworks **Forms I–VII**. We find that all the structures are stabilised significantly by solvent inclusion in their voids; this stabilisation is calculated to be 29.4–133.8 kJ mol^–1^ for the various solvates. Importantly, these large stabilisation energies lower the lattice energy of the known solvates below the hypothetical CSP global minimum energy structure on the crystal energy landscape (Fig. 3). This demonstrates that the solvated **CC1** structures are more stable than the unsolvated CSP global minimum and rationalises why the observed solvated structures of **CC1** are formed from organic solvents in preference to the lowest energy possible packing of pure **CC1**. The extent of the calculated solvent stabilisation with respect to the CSP global minimum ranges from 13.3 (**Form VIII**, *p*X) to 77.1 (**Form III**, CCl_4_) kJ mol^–1^.

The data in Fig. 4 also show how the **CC1** solvate system lowers its energy quickly as the first solvent molecules are added, but then starts to level off, approaching a minimum in stabilisation energy as more solvent is added (for DCM in **Form I**, around *N* = 3 and 4). Beyond *N* = 4, any further addition of DCM completely disrupts the **CC1 Form I** framework structure. For each solvatomorph, we predict the solvent loading as the composition with the lowest energy before the **CC1** arrangement is disrupted.

For the most stabilised solvent structures, the Monte Carlo predicted solvent loading agrees remarkably well with the experimental composition (Table 1). For all cage-solvent pairings, the predicted ratio of **CC1** to solvent is within 1 of the experimental value, which represents the closest possible agreement, given the use of integer sampling ratios used in the computational work. This suggests that it is possible to predict, *a priori*, the most stable solvation level for a given solvent within a predicted structure on a CSP landscape.

**Table 1:** Observed (*N*_observed_) and calculated (*N*_predicted_) solvent loadings (**CC1** : solvent, 1 : *N*) of the known solvatomorphs of **CC1**

	Solvent	*N*_observed_	*N*_predicted_
**Form I**	DCM	3.88	4
**Form II**	CHCl_3_	5.66	5
**Form III**	CCl_4_	5	5
**Form IV**	EtOAc	2.5	2
**Form V**	1,4-Dioxane	3.5	3
**Form VI**	CHCl_3_·*o*X	1 : 1	1 : 1
**Form VII**	DCM·*o*X	1 : 2	1 : 2
**Form VIII**	*p*X	1.47	1

### Preferential solvent stabilisation between **CC1** frameworks

While the results summarised in Fig. 3 demonstrate that all the solvates tested are indeed predicted to be more stable than the desolvated CSP global minimum structure, they do not answer a key question: is the observed experimental solvatomorph the most stable structure for that specific solvent?

To assess the predictive potential of our methods, we considered the five solvents DCM, CHCl_3_, CCl_4_, EtOAc and 1,4-dioxane, using the Monte Carlo procedure to insert each solvent into each of the artificially desolvated **CC1 Forms I–V**, as well as the CSP global minimum structure (CSP min). The solvated lattice energies were then calculated for the most stable loading of each solvent into each solvate framework (Table 2).

**Table 2:** Comparison of the *E*_Latt_ (kJ mol^–1^) for a selection of different solvated **CC1** crystal packing arrangement (**Forms I–V**) combinations

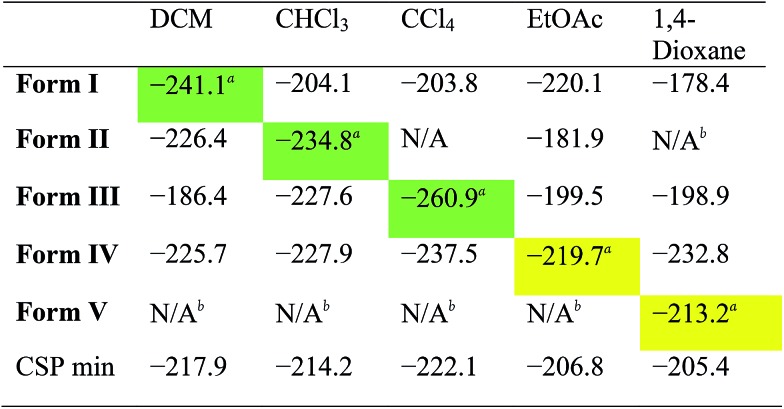

^*a*^Green highlighting indicates that the experimentally determined solvent is the most stabilising; yellow indicates that another solvent is more stabilising.

^*b*^N/A denotes structures into which solvent could not be inserted without significantly distorting the **CC1** packing (see ESI).

First, these results clearly rationalise why the CSP global lattice energy minimum structure is not observed from crystallisation in the presence of any of these five solvents. The global minimum structure from the CSP landscape can accommodate each of the solvents studied here, and it is energetically stabilised by most solvents (final row, Table 2), apart from *o*X and *p*X, where the lattice energy is increased relative to the neat, solvent-free **CC1** structure (Table S8†). However, for all the solvents tested, there is always at least one competing solvate, including the experimentally observed solvate in each case, that is more stable (Tables 2, S8 and S9†); as such, the global minimum predicted structure is never predicted to be favoured.

For the three chlorinated solvents, DCM, CHCl_3_, and CCl_4_, we found excellent agreement between prediction and experiment; for each solvent, the observed solvate structure is the most stable of the **CC1** structures after computational solvation (Table 2, green highlighted values).

For EtOAc, we find that **Form I** and **Form IV** (the observed EtOAc solvatomorph) are effectively equi-energetic, with **Form I** being slightly more stable by 0.4 kJ mol^–1^. EtOAc is the largest and most flexible of the solvents tested in this cross comparison and it is possible that the rigid-molecule approach used in our calculations performs less well in terms of distinguishing the observed solvate structure than for essentially rigid solvent molecules (*i.e.*, DCM, CHCl_3_, and CCl_4_). Nonetheless, from these calculations, one might predict that EtOAc would lead to either **Form I** or **Form IV**, the latter of which is observed by experiment.

In the case of the 1,4-dioxane solvate, we found that none of the four alternative solvents could solvate the **CC1** framework of the observed structure (**Form V**); for each of DCM, CHCl_3_, CCl_4_ and EtOAc, solvent insertion leads to a rearrangement of the **Form V CC1** framework. The observed packing, in which 1,4-dioxane lies flat between the cages, cannot be replicated by the other solvents. The fact that no other solvent can form this solvated crystal structure agrees well with experiment – in a previous study,^23^ more than 40 other solvents were trialled in the laboratory, but none was found to direct the **CC1** window-to-window packing observed in the 1,4-dioxane solvate, **Form V**. Comparing the inclusion of 1,4-dioxane within the set of **CC1** frameworks (column 5; Table 2), we find that 1,4-dioxane forms a more stable solvate in the **Form IV CC1** arrangement than the observed **Form V**. This is due to a failure of the Monte Carlo sampling to locate the most stable configuration of 1,4-dioxane within **Form V**; by calculating the lattice energy using the positions of 1,4-dioxane from the experimentally determined crystal structure, we obtain an energy for the **Form V** 1,4-dioxane solvate of –241.9 kJ mol^–1^. This configuration is 28.7 kJ mol^–1^ more stable than the most stable configuration determined by our solvent insertion methodology and it renders **Form V** the lowest energy of the tested pairings between 1,4-dioxane solvent and the **CC1** host frameworks. This 1,4-dioxane result shows that the solvent filling approach could be successful, but illustrates that further developments in solvent configuration sampling methods are needed to make these methods more robust in the future.

Solvent insertion into predicted crystal structures shows good results in terms of qualitatively identifying those crystal structures in a CSP landscape that can be solvent stabilised and assessing the preferential solvent stabilisation between possible host frameworks. One aspect that we are as yet unable to address with the current method is the possibility of structural transformations that can occur upon desolvation of a solvated crystal structure. For example, desolvation of **CC1**·2.5(EtOAc) to afford **CC1**-α′ results in a 10% contraction of the unit cell volume and a transformation of the space group from *C*2/*c* to *P*2_1_/*c*.^22^ Artificial, *in silico* desolvation and lattice energy minimisation of the experimental **CC1**·2.5(EtOAc) solvate leads to a polymorph which is 6.3 kJ mol^–1^ higher on the CSP landscape than the experimentally determined structure. Likewise, the DCM solvate, 2(**CC1**)·7.75(DCM), transforms during desolvation which results in a change from 2 to 1 molecule in the asymmetric unit (*Z*′ = 2 → *Z*′ = 1), which is related to a conformational change in one of the **CC1** molecules.^22^

### Comparison with thermogravimetric analysis

To further validate the results of our computational methods, we compared the magnitudes of the calculated solvent stabilisation energies with an experimentally determined measure of the solvate stability. Specifically, we assessed the thermal stability of each single solvate by measuring the difference (*T*_onset_ – *T*_bp_) by thermogravimetric analysis (TGA), where *T*_onset_ and *T*_bp_ are the temperatures that mark the onset of guest release and the boiling point of the guest solvent, respectively (Table S10 and Fig. S15†). This quantity, (*T*_onset_ – *T*_bp_), has been used previously to probe thermal stability of host-guest systems, including clathrates.^44^,^45^

**Fig. 5:**
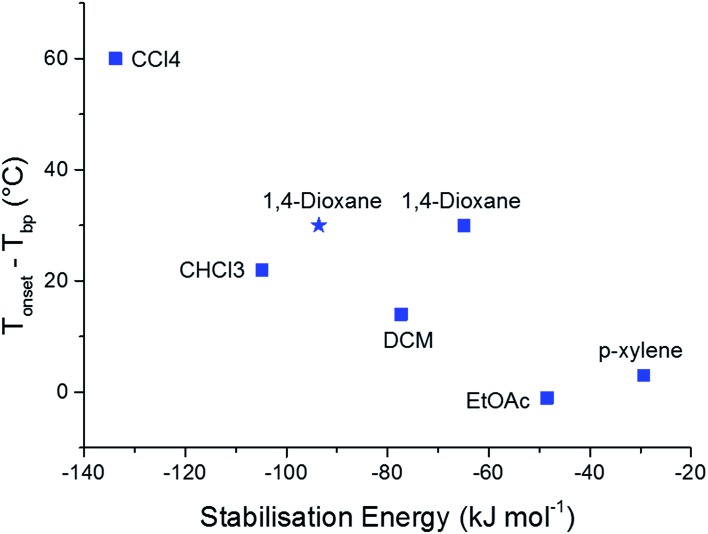
Correlation between (*T*_onset_ – *T*_bp_) and the calculated solvent stabilisation energy for the observed **CC1** solvates. The blue squares show the lowest energies calculated from the Monte Carlo simulations. The calculated stabilisation energy from the lattice energy minimised experimental structure of the 1,4-dioxane solvate is also shown as a blue star.

A broad correlation is observed between (*T*_onset_ – *T*_bp_) and the calculated solvent stabilisation energies (Fig. 5). TGA shows that 2(**CC1**)·10(CCl_4_) is by far the most thermally stable of the **CC1** solvates (*T*_onset_ – *T*_bp_ = 60 °C) and this solvate also has the largest calculated stabilisation energy (–133.8 kJ mol^–1^). This encouraging comparison extends to all three chlorinated solvents and EtOAc, where the stability orders based on TGA and the computational results are the same. The 1,4-dioxane solvate also fits this correlation, particularly when we use the energy calculated using the experimentally-determined solvent position within the **Form V CC1** framework (blue star in Fig. 5), rather than the Monte Carlo minimum energy solvent configuration.

## Conclusions

In summary, we have shown how a Monte Carlo solvent insertion procedure can be used to rationalise the stabilities of the different solvated crystal structures of a highly solvatomorphic porous organic cage, **CC1**. All the observed solvated structures, with eight different crystal packing motifs, are stabilised significantly with respect to their corresponding desolvated **CC1** framework by up to 133.8 kJ mol^–1^. As a result, the observed solvates are calculated to be more energetically stable than the lowest energy **CC1** crystal structure calculated using crystal structure prediction methods. Thus, we rationalise why these relatively high energy **CC1** crystal packing arrangements are observed, rather than the predicted global minimum, when this molecule is crystallised from solvent. This presents a challenge for CSP by demonstrating that experimentally relevant crystal structures can occupy high energy regions on the landscape, so that crystal structure search algorithms should have the capability to provide complete structure sets, even high above the global lattice energy minimum.

This computational approach is also shown to have promise in predicting the preferential stabilisation that different solvents provide to different porous crystal structures: for example, the calculations explain why three structurally-similar chlorinated solvents force **CC1** to adopt three different solvated crystal packing motifs. In each case, the solvated structure is most stable in the correct experimental packing motif and that packing motif is stabilised the most by the solvent from which it is crystallised.

These calculations could be used in a predictive sense by solvating the crystal structures produced by CSP with a range of solvents to identify a solvent that might be used to preferentially stabilise a targeted porous packing arrangement. Such an approach would benefit from a more efficient global optimiser to find the lowest energy solvent configuration within each framework, especially given the failure in this work to locate the most favourable 1,4-dioxane configuration within the **Form V** structure. The assumption of ordered solvent within porous crystal structures should also be relaxed in future developments of the method; where multiple, energetically similar solvent configurations are possible, the solvent is likely to be disordered and the related entropic stabilisation should be considered. If the target is to desolvate the resulting structure to create a porous material, the methods should also be developed to assess the possible changes in the crystal packing motif that may occur upon desolvation, as seen in the conversion of **CC1**-α to **CC1**-α′.^22^

We hope that this study will motivate further development in these methods, which could have an important impact on the *in silico* prediction of solvated structures and, hence, the *a priori* design of new porous molecular crystals.

## Conflicts of interest

There are no conflicts to declare.

## Supplementary Material

Supplementary information

Supplementary information

Supplementary information

Supplementary information

Crystal structure data

## References

[cit1] Day G. M. (2011). Crystallogr. Rev..

[cit2] Price S. L. (2014). Chem. Soc. Rev..

[cit3] Kazantsev A. V., Karamertzanis P. G., Adjiman C. S., Pantelides C. C., Price S. L., Galek P. T. A., Day G. M., Cruz-Cabeza A. J. (2011). Int. J. Pharm..

[cit4] Neumann M. A., van de Streek J., Fabbiani F. P. A., Hidber P., Grassmann O. (2015). Nat. Commun..

[cit5] Braun D. E., McMahon J. A., Koztecki L. H., Price S. L., Reutzel-Edens S. M. (2014). Cryst. Growth Des..

[cit6] Della Valle R. G., Venuti E., Brillante A., Girlando A. (2003). J. Chem. Phys..

[cit7] Campbell J. E., Yang J., Day G. M. (2017). J. Mater. Chem. C.

[cit8] Yang Y., Rice B., Shi X., Brandt J. R., Correa da Costa R., Hedley G. J., Smilgies D.-M., Frost J. M., Samuel I. D. W., Otero-de-la-Roza A., Johnson E. R., Jelfs K. E., Nelson J., Campbell A. J., Fuchter M. J. (2017). ACS Nano.

[cit9] Musil F., De S., Yang J., Campbell J. E., Day G. M., Ceriotti M. (2018). Chem. Sci..

[cit10] Pulido A., Chen L., Kaczorowski T., Holden D., Little M. A., Chong S. Y., Slater B. J., McMahon D. P., Bonillo B., Stackhouse C. J., Stephenson A., Kane C. M., Clowes R., Hasell T., Cooper A. I., Day G. M. (2017). Nature.

[cit11] Evans J. D., Huang D. M., Haranczyk M., Thornton A. W., Sumby C. J., Doonan C. J. (2016). CrystEngComm.

[cit12] Pyzer-Knapp E. O., Thompson H. P. G., Schiffmann F., Jelfs K. E., Chong S. Y., Little M. A., Cooper A. I., Day G. M. (2014). Chem. Sci..

[cit13] Jones J. T. A., Hasell T., Wu X., Bacsa J., Jelfs K. E., Schmidtmann M., Chong S. Y., Adams D. J., Trewin A., Schiffman F., Cora F., Slater B., Steiner A., Day G. M., Cooper A. I. (2011). Nature.

[cit14] Navrotsky A., Mazeina L., Majzlan J. (2008). Science.

[cit15] Barnard A. S., Curtiss L. A. (2005). Nano Lett..

[cit16] Sun W., Jayaraman S., Chen W., Persson K. A., Ceder G. (2015). Proc. Natl. Acad. Sci. U. S. A..

[cit17] Belenguer A. M., Lampronti G. I., Cruz-Cabeza A. J., Hunter C. A., Sanders J. K. M. (2016). Chem. Sci..

[cit18] Stöger B., Kautny P., Lumpi D., Zobetz E., Fröhlich J. (2012). Acta Crystallogr., Sect. B: Struct. Sci..

[cit19] Aakeröy C. B., Champness N. R., Janiak C., Champness R., Janiak C. (2010). CrystEngComm.

[cit20] Karpinska J., Erxleben A., McArdle P. (2011). Cryst. Growth Des..

[cit21] Nassimbeni L. R. (2003). Acc. Chem. Res..

[cit22] Jones J. T. A., Holden D., Mitra T., Hasell T., Adams D. J., Jelfs K. E., Trewin A., Willock D. J., Day G. M., Bacsa J., Steiner A., Cooper A. I. (2011). Angew. Chem., Int. Ed..

[cit23] Hasell T., Culshaw J. L., Chong S. Y., Schmidtmann M., Little M. A., Jelfs K. E., Pyzer-Knapp E. O., Shepherd H., Adams D. J., Day G. M., Cooper A. I. (2014). J. Am. Chem. Soc..

[cit24] Mastalerz M., Schneider M. W., Oppel I. M., Presly O. (2011). Angew. Chem., Int. Ed..

[cit25] Jelfs K. E., Wu X., Schmidtmann M., Jones J. T. A., Warren J. E., Adams D. J., Cooper A. I. (2011). Angew. Chem..

[cit26] Cruz-Cabeza A. J., Day G. M., Jones W. (2009). Chem. - Eur. J..

[cit27] Florence A. J., Johnston A., Price S. L., Nowell H., Kennedy A. R., Shankland N. (2006). J. Pharm. Sci..

[cit28] Tozawa T., Jones J. T. A., Swamy S. I., Jiang S., Adams D. J., Shakespeare S., Clowes R., Bradshaw D., Hasell T., Chong S. Y., Tang C., Thompson S., Parker J., Trewin A., Bacsa J., Slawin A. M. Z., Steiner A., Cooper A. I. (2009). Nat. Mater..

[cit29] Hasell T., Wu X., Jones J. T. A., Bacsa J., Steiner A., Mitra T., Trewin A., Adams D. J., Cooper A. I. (2010). Nat. Chem..

[cit30] Nowell H., Barnett S. A., Christensen K. E., Teat S. J., Allan D. R. (2012). J. Synchrotron Radiat..

[cit31] Sheldrick G. M., SADABS Programs Scaling Absorpt. Correct. Area Detect. Data, University of Göttingen, Göttingen, Germany, 1997.

[cit32] Sheldrick G. M. (2010). Acta Crystallogr., Sect. D: Biol. Crystallogr..

[cit33] Sheldrick G. M. (2008). Acta Crystallogr., Sect. A: Found. Crystallogr..

[cit34] Sheldrick G. M. (2015). Acta Crystallogr., Sect. C: Struct. Chem..

[cit35] Cohelo A., Coelho Software (v. 5), TOPAS-Academic, Brisbane, Australia, 2012.

[cit36] Case D. H., Campbell J. E., Bygrave P. J., Day G. M. (2016). J. Chem. Theory Comput..

[cit37] Price S. L., Leslie M., Welch G. W. A., Habgood M., Price L. S., Karamertzanis P. G., Day G. M. (2010). Phys. Chem. Chem. Phys..

[cit38] Williams D. E. (2001). J. Comput. Chem..

[cit39] Stone A. J., Alderton M. (1985). Mol. Phys..

[cit40] Martin M. G. (2013). Mol. Simul..

[cit41] Chisholm J. A., Motherwell S. (2005). J. Appl. Crystallogr..

[cit42] Slater A. G., Reiss P. S., Pulido A., Little M. A., Holden D. L., Chen L., Chong S. Y., Alston B. M., Clowes R., Haranczyk M., Briggs M. E., Hasell T., Day G. M., Cooper A. I. (2017). ACS Cent. Sci..

[cit43] Nyman J., Day G. M. (2015). CrystEngComm.

[cit44] Atwood J. L., Barbour L. J., Jerga A. (2002). Science.

[cit45] Kane C. M., Banisafar A., Dougherty T. P., Barbour L. J., Holman K. T. (2016). J. Am. Chem. Soc..

