# Supplementary material for: Computational modelling of solvent effects in a prolific solvatomorphic porous organic cage
Source: Faraday Discuss. 2018 Apr 16;211(0):383–99. doi: 10.1039/c8fd00031j (PMC6208051; doi:10.1039/c8fd00031j)
Supplement: Supplementary file 1 [file FD-211-C8FD00031J-s001.pdf]

Electronic Supplementary Material (ESI) for Faraday Discussions.
This journal is © The Royal Society of Chemistry 2018

Supplementary information:

Computational Modelling of Solvent Effects in a Prolific Solvatomorphic Porous Organic Cage

David P. McMahon,[a] Andrew Stephenson,[b] Samantha Y. Chong,[b] Marc A. Little,[b] James T. A. Jones,[b,c] Andrew I. Cooper*,[b] and Graeme M. Day*,[a]

[a.] Computational Systems Chemistry, School of Chemistry, University of Southampton, SO17 1BJ, UK

[b.] Department of Chemistry and Materials Innovation Factory, University of Liverpool, Crown St., Liverpool L69 7ZD, UK

[c.] Present Address: Defence Science and Technology Laboratory, Dstl, Porton Down, Salisbury, Wiltshire U.K.

Materials

All solvents were purchased from Sigma-Aldrich and used as received. 1,3,5-Triformylbenzene was purchased from Manchester Organics, UK.

Synthesis and Crystallisation

The ethylene cage (**CC1-*β*'**) was prepared using the scaled up procedure reported previously[1] and used in all crystallisation experiments in its desolvated form. **CC1-*α*** could be crystallised from DCM/EtOAc or $CHCl_3$/EtOAc with a solvent ratio of 1:2 v/v. **CC1** (168 mg, 2.12 x10^{-4} mol) was dissolved in DCM or $CHCl_3$ (28 ml) and layered onto EtOAc (56 ml) to give clear needle type crystals after 4-6 weeks.

CC1·CCl_4 was prepared by dissolving **CC1** (40 mg, 5.05 x10^{-5} mol) in $CHCl_3$ (5 ml), CCl_4 (6 ml) was slowly layered onto the mixture which was slowly evaporated under a N_2 flow to give clear cubic crystals (3 - 5 days).

CC1·$CHCl_3$ was crystallised by dissolving **CC1** in $CHCl_3$ and allowing the solvent to evaporate very slowly. Crystals were kept as solvated as possible by ensuring that there was still $CHCl_3$ in the vial when they were collected. **CC1·DCM** was prepared using the analogous procedure but with DCM as the solvent.

CC1·pX was prepared by an analogous procedure to CC1-γ as reported previously.[1]

CC1·$CHCl_3$/oX was prepared using a H-tube with **CC1** (20 mg, 2.53 x10^{-5} mol) dissolved in $CHCl_3$ (3 ml) in one side and o-Xylene (3.5 ml) in the other. This was left sealed for 2 -3 weeks to give white needle like crystals. **CC1·DCM/oX** was prepared using an analogous procedure.

Single crystal X-ray crystallography

Table S1. Single crystal X-ray refinement details for 2(**CC1**)·7.75(CH_2Cl_2), 2(**CC1**)·11.32($CHCl_3$) and 2(**CC1**)·10(CCl_4).

Sample Reference	2(**CC1**)·7.75(CH_2Cl_2)	2(**CC1**)·11.32($CHCl_3$)	2(**CC1**)·10(CCl_4)
Collection Temperature	100 K	150 K	100 K
λ [Å]	0.6889	0.71073	0.71073
Formula	$C_{103.75}H_{111.5}Cl_{15.5}N_{24}$	$C_{107.33}H_{107.33}Cl_{33.97}N_{24}$	$C_{106}H_{96}Cl_{40}N_{24}$
Mr	2244.14	2937.63	3124.06
Crystal Size (mm)	0.17 x 0.15 x 0.15	0.37 x 0.20 x 0.20	0.18 x 0.15 x 0.08
Crystal System	Trigonal	Triclinic	Cubic
Space Group	*R*3	$P\bar{1}$	*F*23
a [Å]	21.3987(16)	17.1487(11)	24.3478(5)
b [Å]	-	18.3913(11)	-
c [Å]	21.3164(18)	24.7171(15)	-
α [°]	-	73.378(2)	-
β [°]	-	72.980(2)	-
γ [°]	-	76.700(2)	-
V [Å^3]	8453.2(15)	7050.9(8)	14433.7(9)
Z	3	2	8
D_{calcd} [g cm^{-3}]	1.323	1.384	1.438
μ[mm^{-1}]	0.393	0.704	0.800
F(000)	3496	2994	6320
2ϑ range [°]	2.822 – 51.000	1.772 – 52.764	3.346 – 54.854
Reflections collected	30840	117695	36034
Independent reflections, R_{int}	7407, 0.0721	28822, 0.0499	2780, 0.0411
Obs. Data [$I > 2\sigma$]	6606	22595	2404
Data / restraints / parameters	7407 / 9 / 471	28822 / 199 / 1707	2780 / 0 / 174
Final $wR(F^2)$ values ($I > 2\sigma(I)$)	0.0912	0.0798	0.0468
Final *R*1 values (all data)	0.0973	0.0967	0.0551
Final $wR(F^2)$ values (all data)	0.2591	0.2512	0.1428
Goodness-of-fit on F^2	1.065	1.038	1.046
Largest diff. peak and hole [e.A-3]	1.116 / -1.354	1.530 / -0.979	0.282 / -0.166
Flack parameter	0.33(3)	-	-0.14(2)
CCDC	1575913	1575914	1575912

Table S2. Single crystal X-ray refinement details X-ray refinement details for **CC1**·2(o-xylene)·$CHCl_3$ and **CC1**·o-xylene·CH_2Cl_2.

Sample Reference	**CC1**·2(o-xylene)·$CHCl_3$	**CC1**·o-xylene·CH_2Cl_2
Collection Temperature	100 K	100 K
λ [Å]	0.7749	0.6889
Formula	$C_{65}H_{69}Cl_3N_{12}$	$C_{57}H_{60}Cl_2N_{12}$
Mr	1124.67	984.07
Crystal Size (mm)	0.07 x 0.01 x 0.01	0.08 x 0.01 x 0.005
Crystal System	Orthorhombic	Monoclinic
Space Group	$P2_12_12_1$	*P*21/*n*
a [Å]	10.654(3)	16.565(4)
b [Å]	23.151(6)	10.745(3)
c [Å]	25.363(7)	31.067(8)
β [°]		92.088(6)
V [Å^3]	6256(3)	5526(2)
Z	4	4
D_{calcd} [g cm^{-3}]	1.194	1.183
μ[mm^{-1}]	0.243	0.151
F(000)	2376	2080
2ϑ range [°]	2.596 – 45.584	3.888 – 40.294
Reflections collected	22631	12268
Independent reflections, R_{int}	6517, 0.1306	5628, 0.0880
Data / restraints / parameters	6517 / 0 / 752	5628 / 527 / 660
Final $wR(F^2)$ values ($I > 2\sigma(I)$)	0.0654	0.0958
Final *R*1 values (all data)	0.1079	0.2278
Final $wR(F^2)$ values (all data)	0.1596	0.2865
Goodness-of-fit on F^2	1.005	0.986
Largest diff. peak and hole [e.A-3]	0.198 / -0.241	0.574 / -0.512
Flack parameter	0.30(10)	
CCDC	1575911	1575910

Refinement notes and crystal structure plots:

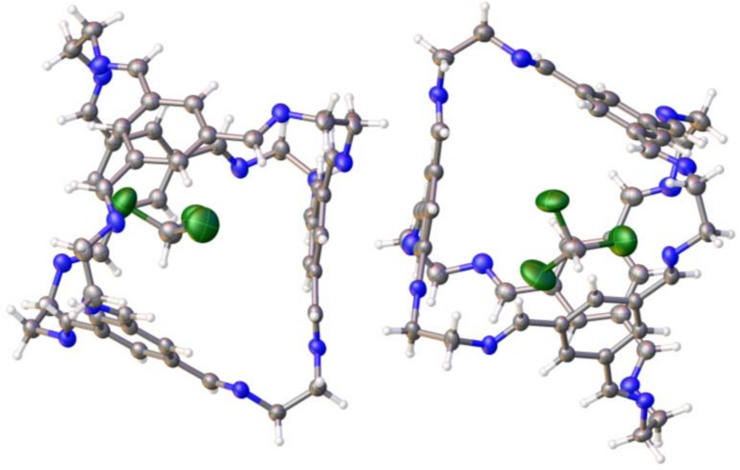

Figure S1. Displacement ellipsoid plot from the single crystal structure, 2(**CC1**)·11.32($CHCl_3$), only ordered $CHCl_3$ molecules, that are located in the **CC1** cavity, are shown for clarity. Ellipsoids displayed at 50 % probability level.

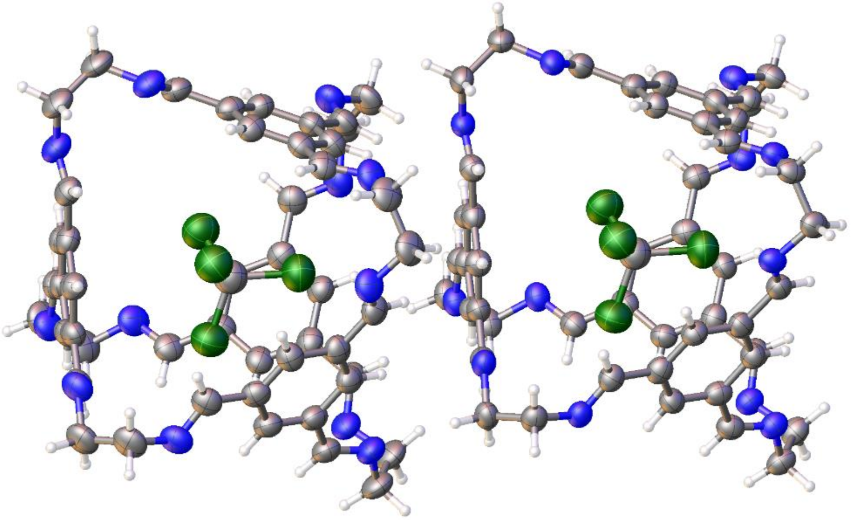

Figure S2. Displacement ellipsoid plot from the single crystal structure, 2(**CC1**)·10(CCl_4), **CC1** and CCl_4 molecules shown in entirety. Ellipsoids displayed at 50 % probability level; disordered CCl_4 omitted for clarity.

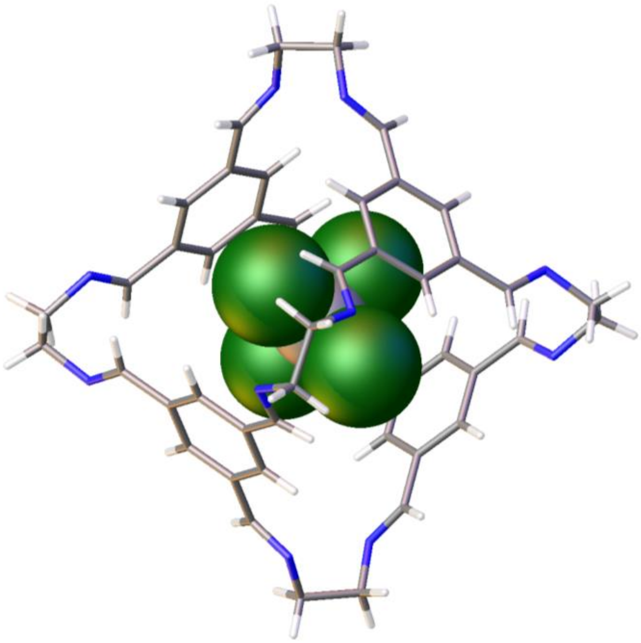

Figure S3. Orientation of ordered CCl_4 in **CC1** cavity in the single crystal structure, 2(**CC1**)·10(CCl_4).

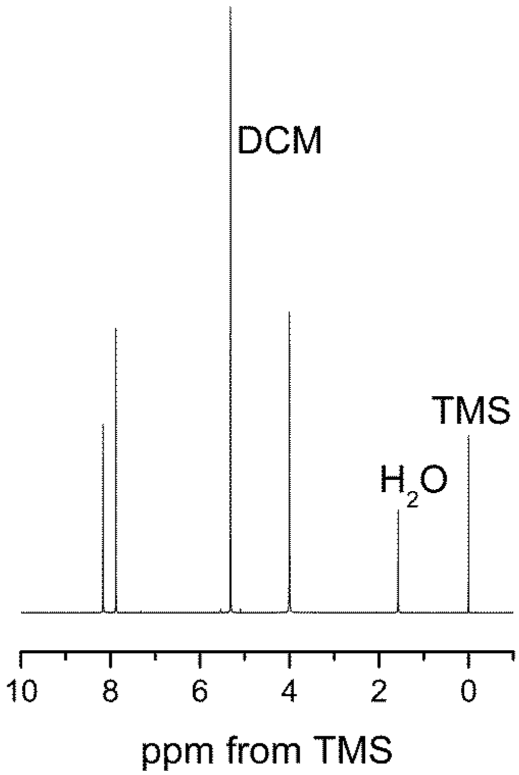

Figure S4. ^{1}H NMR (400 MHz, CD_2Cl_2) spectra of as crystallised 2(**CC1**)·10(CCl_4).

Refinement notes for, 2(**CC1**)·7.75(CH_2Cl_2):

One CH_2Cl_2 molecules, positioned in a **CC1** cavity, was severely disordered and refined with bond distance restraints (DFIX in SHELX) and constrained displacement parameters (EADP in SHELX). For a second disordered CH_2Cl_2 molecule the C-atom was refined with restrained displacement parameters (ISOR in SHELX). For a displacement ellipsoid plot, see **Figure S5**.

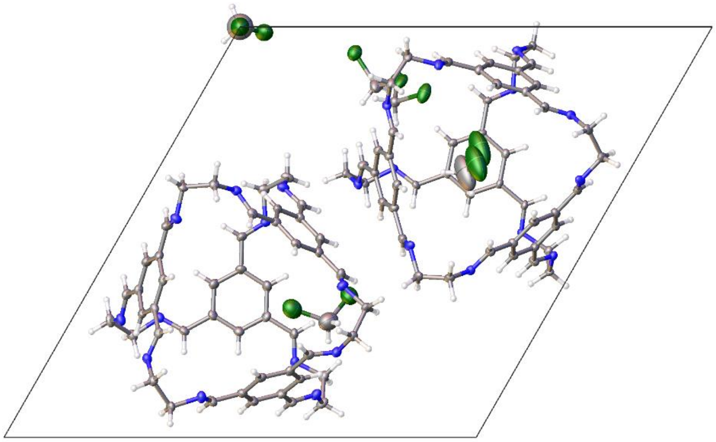

Figure S5. Displacement ellipsoid plot from the single crystal structure, 2(**CC1**)·7.75(CH_2Cl_2), **CC1** molecules shown in entirety; perspective view [001]; ellipsoids displayed at 50 % probability level.

Refinement notes for, **CC1**·2(o-xylene)·$CHCl_3$:

Due to disorder, high angle X-ray diffraction was weak, despite using synchrotron radiation. A suitable resolution limit of 1.0 Å resolution limit was applied during refinement. One $CHCl_3$, located in the **CC1** cavity, was modelled over two positions, for each $CHCl_3$ molecule, the C-atoms was refined isotropically. For a displacement ellipsoid plot, see **Figure S6**, and for responses to the A- and B- checkCIF alerts, see below:

_THETM01_ALERT_3_A
Problem: The value of sine(theta_max)/wavelength is less than 0.550 Calculated sin(theta_max)/wavelength = 0.4999.
Response: Due to disorder, X-ray diffraction was weak, despite using synchrotron radiation. A suitable resolution limit of 1.0 ang. was applied during refinement.

_PLAT089_ALERT_3_B
Problem: Large U3/U1 Ratio for Average U(i,j) Tensor .... 4.8 Note
Response: Due to disorder, X-ray diffraction was weak, despite using synchrotron radiation. A suitable resolution limit of 1.0 ang. was applied during refinement and the number of unique reflections is lower than expected.

_PLAT089_ALERT_3_B
Problem: Low Bond Precision on C-C Bonds .... 0.01447 Ang.
Response: Due to disorder and the limited resolution of the X-ray data.

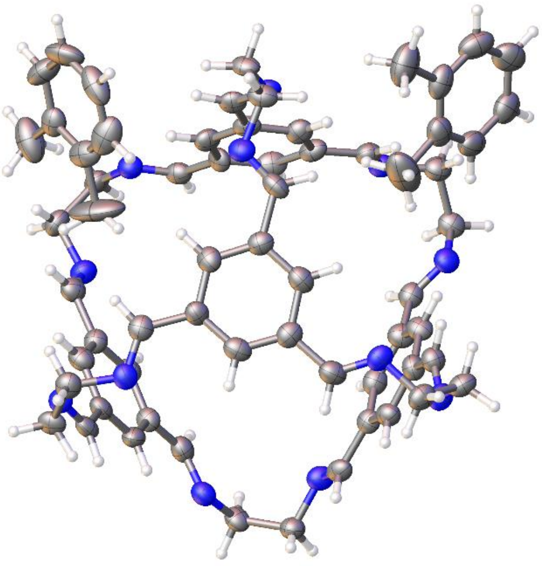

Figure S6. Displacement ellipsoid plot from the single crystal structure, **CC1**·2(o-xylene)·($CHCl_3$), disordered $CHCl_3$, located in the **CC1** cavity, omitted for clarity; ellipsoids displayed at 50 % probability level.

Refinement notes for, **CC1**·2(o-xylene)·$CHCl_3$:

During refinement of the structure a 1.0 Å resolution limit was applied, and a group rigid-bond restraint was used (RIGU in SHELX). One CH_2Cl_2 molecule, located in the **CC1** cavity, was modelled over two positions, 1,2 and 1,3 bond distance restraints were used during refinement (DFIX and DANG in SHELX). For a displacement ellipsoid plot, see **Figure S7**, and for responses to the A- and B- checkCIF alerts, see below:

_THETM01_ALERT_3_A
Problem: The value of sine(theta_max)/wavelength is less than 0.550 Calculated sin(theta_max)/wavelength = 0.4999
Response: Due to disorder, X-ray diffraction was weak, despite using synchrotron radiation. A suitable resolution limit of 1.0 ang. was applied during refinement.

_PLAT089_ALERT_3_B
Problem: Large U3/U1 Ratio for Average U(i,j) Tensor .... 4.8 Note
Response: Due to disorder, X-ray diffraction was weak, despite using synchrotron radiation. A suitable resolution limit of 1.0 ang. was applied during refinement and the number of unique reflections is lower than expected.

_PLAT089_ALERT_3_B
Problem: Low Bond Precision on C-C Bonds .... 0.01447 Ang.
Response: Due to disorder and the limited resolution of the X-ray data.

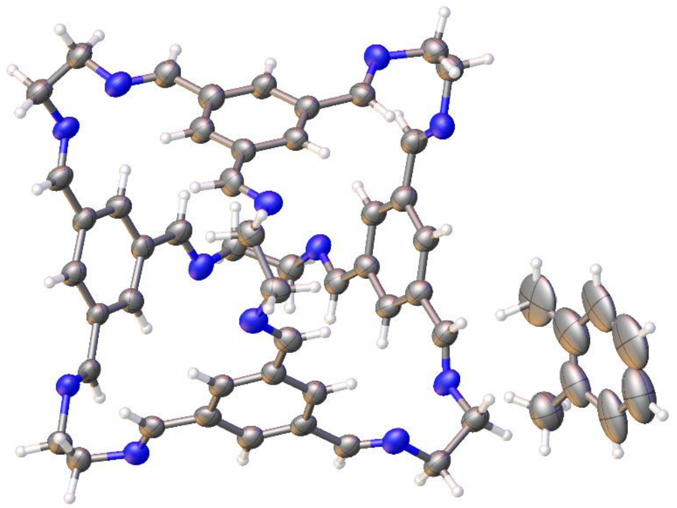

Figure S7. Displacement ellipsoid plot from the single crystal structure, **CC1**·(o-xylene)·(CH_2Cl_2), disordered CH_2Cl_2, located in the **CC1** cavity, omitted for clarity; ellipsoids displayed at 50 % probability level.

Powder X-ray diffraction

High resolution synchrotron X-ray diffraction data were collected at beamline I11 at Diamond Light Source, Didcot, Oxfordshire. Data were collected using the position sensitive Mythen-II detector on a sample of **CC1** loaded with *p*-xylene contained in a sealed 0.5 mm diameter borosilicate glass capillary. The capillary was spun during data collection to improve powder averaging.

Indexing was performed using *TOPAS-Academic*,[2] which suggested a monoclinic cell, with the space group $P2_1$ tentatively assigned. A *C*-centred monoclinic cell was also possible, but could not be distinguished after Le Bail fitting the available data. Structure solution was attempted using simulated annealing implemented in *TOPAS-Academic*.[2] The model consisted of the full CC1 molecule and two p-xylene guests occupancies varied during the optimisation. Two molecules were allowed to translate and rotate freely throughout the unit cell; the y-position of the third molecule was fixed, but the position and orientation otherwise allowed to vary freely. No torsion angles were varied during the structure solution calculation. The simulated annealing calculation was run five times and the best solution selected for Rietveld refinement. All five solutions were similar in terms of the positions of the cage and guest molecules, with small variations in the orientation of the *p*-xylene guests.

Rietveld refinement was performed with geometric restraints applied to all bond lengths and angles within the cage molecule and the xylene guests specified as rigid bodies with variable positions, orientations and occupancies. Hydrogen atoms were modelled at standard geometries and refined using the riding model. Hydrogen atoms were not included for the freely rotating methyl groups on the xylene molecules. There are two guest sites in the structure: an extrinsic sites in which the p-xylene guest is positioned with its methyl substituents close to the windows of two cages, and an intrinsic site inside the cage molecule. The extrinsic xylene consistently refined to close to full occupancy, so it was fixed at unity. The xylene guest inside the cage has a much lower refined occupancy of 0.471(2). The isotropic displacement parameters for both guest molecules are large (B_{iso}(Carbon) = 7-11) suggesting that the xylene molecules are poorly ordered at room temperature.

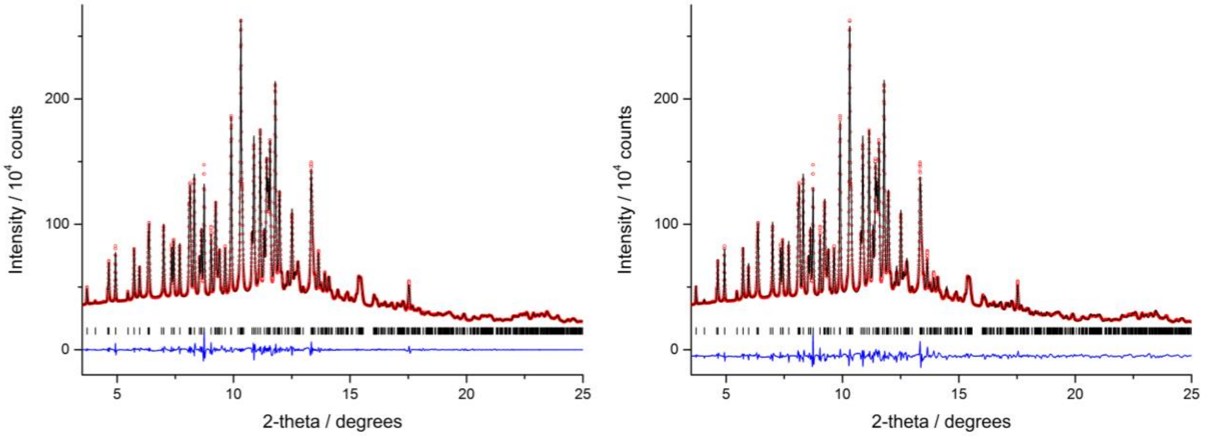

Figure S8. Final observed (red circles), calculated (black line) and difference (blue line) profiles for (left) Le Bail (R_{wp} = 1.82 %, R_p = 1.26 %, ^{2} = 12.3) and (right) Rietveld (R_{wp} = 2.55 %, R_p = 1.91 %, ^{2} = 15.1, R_{Bragg} = 2.95 %; 5376 observations, 492 reflections, 237 parameters, 154 restraints) refinement for **CC1**·1.47(*p*-xylene) (= 0.827157 Å). Reflection positions are marked.

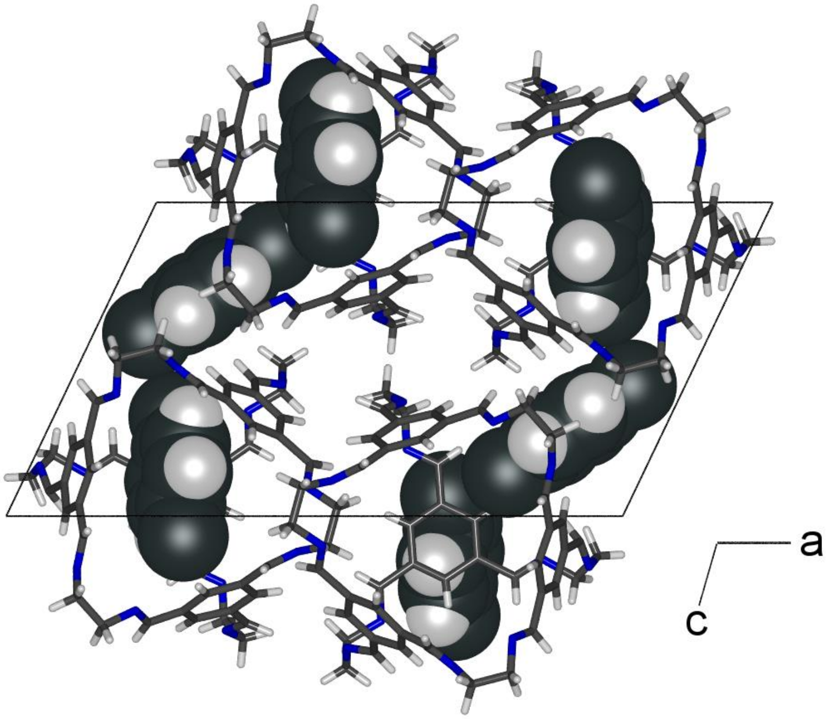

Figure S9. Crystal structure of **CC1**·1.47(*p*-xylene) determined from powder X-ray diffraction. The *p*-xylene guest located inside the cage has a refined partial occupancy of 0.471(2). However, the large isotropic displacement parameters for both guests suggest the xylene positions are poorly ordered.

Thermogravimetric Analysis

TGA was carried out using a TA Q5000IR analyser with an automated vertical overhead thermobalance. Samples were heated at a rate of 10 °C/min.

Experimental solvent stabilisation values have been determined using TGA. The solvates were made using a 'vial in vial' technique. 10 mgs of activated CC1 was placed in an open vial, this was then placed in a larger vial with the appropriate solvent in, sealed and left at room temperature for 3 days. The TGA experiments were run immediately when the material was removed from the vial to minimise solvent loss before the analysis.

T_{onset} was calculated using the in-built feature in the software 'TA Universal Analysis'. The TGA curves and calculated T_{onset} values are shown in Figure S15.

Computational Methods

Crystal Structure Prediction (CSP)

CSP calculations taken from the Z'=1 full search set of ref [3] on the T_d conformer of **CC1** were used to determine the likely crystal packing arrangements of **CC1** in space groups *P*1, $P2_1$, *C*2, $P2_12_12_1$, $P2_12_12$, $C222_1$, $P4_12_12$, *R*3, $P\bar{1}$, *Cc*, $P2_1/c$, *C*2/*c*, $Pna2_1$, *Pbcn*, *Pbca*, and *Pnma*. The CSP structures were taken and the same molecular geometry as used during the Monte-Carlo calculations was overlaid onto the structures. The structures were lattice energy minimised with an isotropic atom-atom potential using DMACRYS2.1.0. Electrostatic interactions were modeled using an atomic hexadecapole description of the molecular charge distribution taken from a B3LYP/6-31G** density obtained from Gaussian09.[4] Repulsion-dispersion interactions were modeled using the W99 potential.[5] An Ewald summation was used to calculate the charge-charge, charge-dipole, and dipole-dipole interactions; all other intermolecular interactions were subject to a 30 Å cutoff.

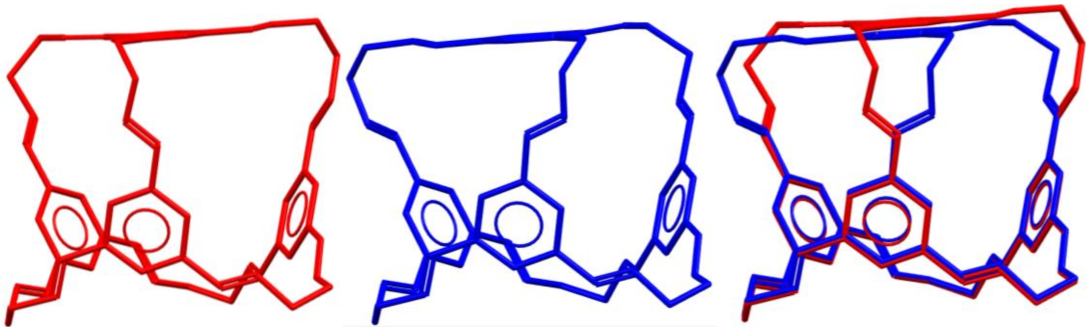

Figure S10: B3LYP/6-31G(d,p) optimised conformations of CC1: T_d (Red), C_3 (Blue) and the overlay of the T_d and C_3 conformers (hydrogens omitted for clarity) with energies of -2514.9965485 and -2514.9899791 Hartree respectively.

For systems in which the desolvated forms of the experimental solvate structures could not be found in the Z'=1 CSP set, additional CSP calculations were performed. This set included the C2/c Z'=1, CC1 Form II ($CHCl_3$); C2/c Z'=2, CC1 Form III (CCl_4) and $P2_1/c$, Z=2 CC1 Form VII (1,4-

Dioxane) which both contained $2 \cdot T_d$ in the asymmetric unit; and finally the P1, Z'=2, CC1 Form I (DCM) which contains one T_d and one C_3 conformer in the asymmetric unit. For these CSP searches, we limited ourselves to only using the experimentally determined space group for each of the given systems. Further to this, a two-step minimisation procedure was used in which a computationally less expensive monopole description of the electrostatics was used, which was obtained, by using MULFIT,[6,7] through fitting atomic partial charges to the molecular electrostatic potential produced from the full multipole model (up to hexadecapoles on each atom). A Lennard-Jones form of the W99 potential (where Lennard-Jones parameters were fitted to reproduce the *exp-6* atom-atom interactions) was used during the first minimisation, during which a small pressure (10 MPa) was applied to help convergence. The pressure was removed and a second minimisation using the Buckingham form of the W99 potential was performed. For the P1, Z'=2 search a total of 10000 valid lattice energy minimisations were performed. For structures higher up the lattice energy landscape (C2/c Z'=1, C2/c Z'=2 and $P2_1/c$ Z=2) a more extensive search of 50000 valid minimisations was tried. The results were clustered using COMPACK to remove duplicates and a further 2 minimisation cycles were performed on the clustered set with hexadecapoles and parameters matching those used to produce the Z'=1 CSP set. The results are collected in Table S3.

Available Volume Calculations

The available volume was calculated using zeo++ with a channel and probe radius of 1.2 Å and 100000 Monte-Carlo samples per unit cell.[8,9]

Monte-Carlo Solvent Insertion Calculations

The experimental CC1 structures were taken, expanded to P1, lattice energy minimised using DMACRYS.[10] Monte Carlo (MC) simulations were performed to determine the role of solvation in stabilising the packing. MC simulations using Towhee-7.10[11] in the NVT ensemble at 5000 K were performed on unit cell systems containing CC1:N Solvent ratio (where N=1, 2, 3 ..) with a force field derived from a combination of ab-initio, UFF[12] and W99[5] parameters with each simulation consisting of 10000 MC cycles using the Monte-Carlo moves of configurational-bias single box molecule reinsertion move (pm1boxcbswap), configurational-bias partial molecule regrowth (pmcb), intramolecular single atom translation move (pmtraat), center-of-mass molecule translation move (pmtracm) and rotation about the center-of-mass move (pmrotate). Additionally, intrabox two molecule switch based upon the center of mass positions (pm1boxcomswitch) was used for systems involving more than one solvent type. Examples of the distribution of move types can be found in Tables S3 and S4.

Table S3: Example solvent Monte-Carlo move types and (where applicable) the target acceptance rates used during the single solvent Monte-Carlo simulations.

Type	Move Distribution (%)	Target Acceptance Rate (%)
pm1boxcbswap	5.8	
pmcb	11.5	50
pmtraat	5.8	50
pmtracm	38.7	25
pmrotate	38.2	25

Table S4: Example solvent Monte-Carlo move types and (where applicable) the target acceptance rates used during the binary solvent Monte-Carlo simulations.

Type	Move Distribution (%)	Target Acceptance Rate (%)
pm1boxcbswap	5.2	
pm1boxcomswitch	10.3	
pmcb	10.4	50
pmtraat	5.2	50
pmtracm	34.7	25
pmrotate	34.2	25

Frames sampled at 10 cycle intervals were used as an input for minimisation with DMACRYS2.1.0 with a 30 Å cutoff for the real-space component of the Ewald summation. Each minimisation cycle consisted of a point charge minimisation using CHELPG charges derived from a Gaussian09 B3LYP/6-31G** calculation,[4] followed by a multipole minimisation with atom-centered multipoles up to the hexadecapole level, derived from a distributed multipole analysis of the B3LYP/6-31G** electron density. A secondary minimisation was performed to ensure the structure was a stable minimum. All valid structures were compared against the desolvated experimental framework using the COMPACK algorithm[13] using molecular clusters consisting of 30 molecules and an interatomic distance tolerance of 20 % in order to eliminate those structures in which there had been a significant change in the CC1 framework ($RMSD_{30}$ > 0.8 Å).

The energy of the solvent ($E_{Solvent}$) was calculated from configurations arising from MC equilibrated, solvent boxes containing 50 molecules fixed at the experimental density. Simulations were in the NVT ensemble (at 300 K) and consisted of 100000 MC cycles with an equal mix of pmtracm and pmrotate moves with a target acceptance rate of 50 %. Frames sampled every 50 MC cycles from the last 50000 MC cycles were subject to a constant volume DMACRYS minimisation with atom-centered multipoles up to the hexadecapole level (derived from a distributed multipole analysis of the B3LYP/6-31G** electron density). Intermolecular interactions were modeled in a similar manner to previous calculations only this time using a 15 Å cutoff. The results of these calculations are collected in Table S5.

$E_{Solvent}$, in conjunction with a factor of (3/2)RT at T=300 K [to account for the change in internal energy of the solvent in the liquid and crystalline state] was used to compute a lattice energy of

CC1 (including the additional stabilisation gained by CC1 through interaction with the solvent) in the solvated system according to equation (1).

$$E_{Latt}=[E_{Latt(CC1+Solvent)}+ E_{CC}+N\times(-E_{Solvent}+(3/2)RT)] \qquad (1)$$

Where E_{CC}=0.5*17.25 kJ mol^{-1}, which is a correction (where applicable) due to the difference in intramolecular energy of the T_d and C_3 CC1 conformations.

Table S5: Solvent constant volume mean energies for 1001 minimisation attempts using frames derived from a Monte-Carlo NVT simulation on a cubic cell containing 50 molecules set to replicate the experimental liquid density.

Solvent	$E_{Latt(Solvent)}$ (kJ / mol)	Std. Dev.	Valid Minimisations
$CHCl_3$	-33.12	0.25	1001
DCM	-31.04	0.20	1001
1,4-Dioxane	-40.13	0.21	1001
EtOAc	-38.84	0.26	1000
p-xylene	-44.43	0.27	999
o-xylene	-45.15	0.25	996
CCl_4	-35.34	0.28	1000

Crystal Structure prediction and solvation calculations

Table S6: Overlay between the experimental structure (with the molecular geometry replaced with the gas-phase optimised structure) and lattice energy minimised desolvated structure.

Structure	N_{Match}/30	RMS
Form I (DCM)	30/30	0.404
Form II ($CHCl_3$)	30/30	1.237
Form V (1,4-dioxane)	23/30	1.561
Form IV (EtOAc)	30/30	0.275
Form III (CCl_4)	30/30	0.525
Form VII (DCM·oX)	30/30	0.587
Form VI ($CHCl_3$·oX)	30/30	0.719
Form VIII (pX)	30/30	0.301

Table S7: Overlay between the lattice energy minimised desolvated experimental structures and the corresponding CSP generated matching structure.

Structure	N_{Match}/30	RMSD N_{Match} (Å)
α′	30/30	0.0083
β′	30/30	0.0025
Form I (DCM)	23/30	0.0017
Form II ($CHCl_3$)	30/30	0.0410
Form III (CCl_4)	30/30	0.0035
Form IV (EtOAc)	30/30	0.0023
Form V (1,4-dioxane)	30/30	0.0045
Form VI ($CHCl_3$·oX)	30/30	0.0048
Form VII (DCM·oX)	30/30	0.0043
Form VIII (pX)	30/30	0.0022

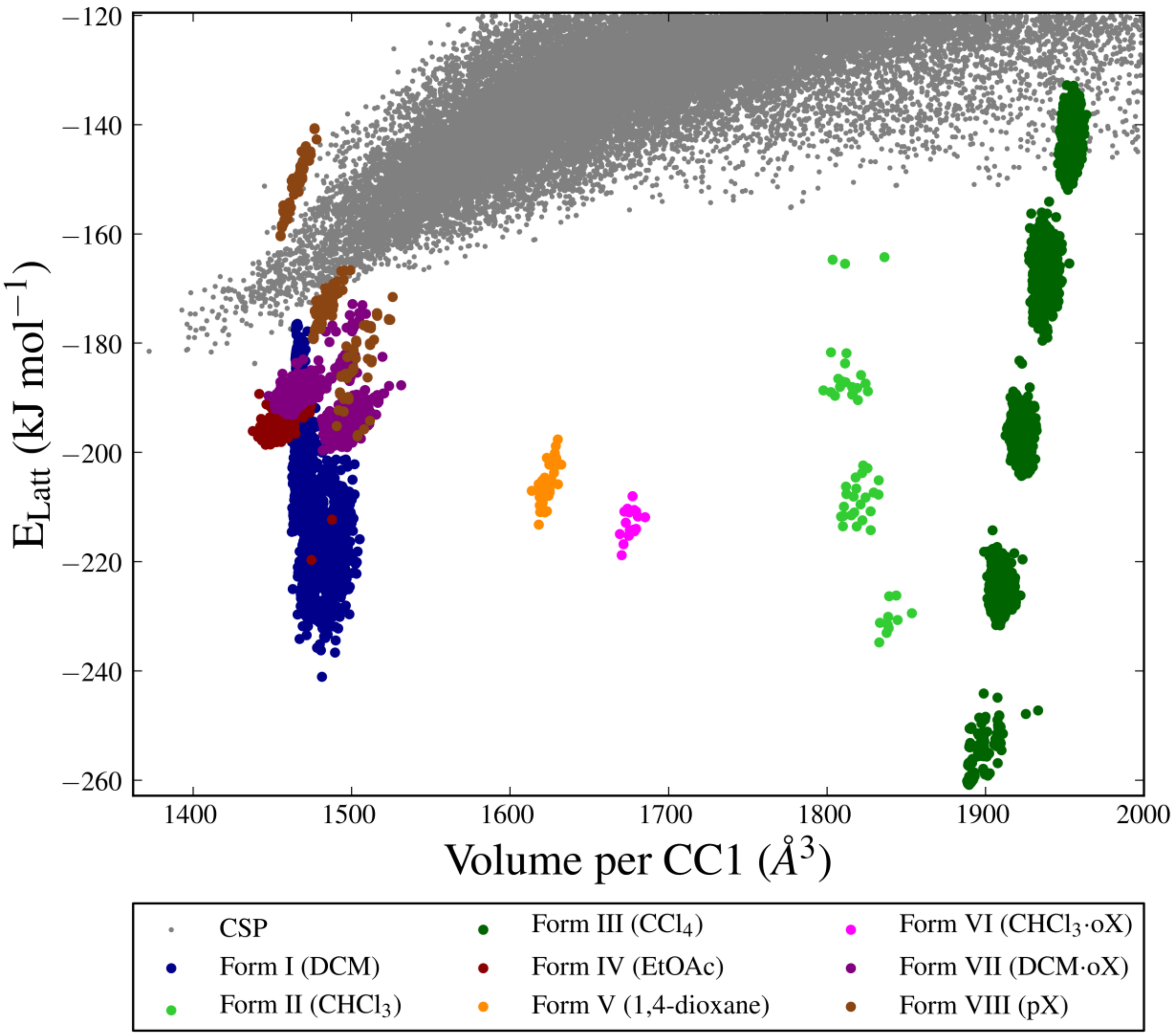

Figure S11. CSP energy landscape for **CC1** (grey points) also showing the calculated latticed energies of the valid solvent stabilised structures (filled circles) for insertion of the solvent into the corresponding experimental form.

Table S8: Data for the lattice energy minimum structure from the solvent insertion into the Z'=1 CSP global minimum structure.

Solvent	E_{Latt} (kJ mol^{-1})	Volume per CC1 (Å^3)	N
Neat	-183.73	1438.89	0
DCM	-217.89	1430.88	3
$CHCl_3$	-214.15	1432.57	2
1,4-dioxane	-205.43	1432.10	2
EtOAc	-206.79	1430.35	2
CCl_4	-222.08	1439.82	2
oX	-183.09	1431.23	2
pX	-170.64	1427.62	2

Table S9: Data for the lattice energy minimum structures from the solvent insertion into the corresponding experimental structure and the corresponding CSP reference points. $E_{Latt(Neat)}$ is the lattice energy of the minimised desolvated structures; $E_{Latt(Solvated)}$ is the lattice energy of the optimal solvated structure; E_{CSP} is the lattice energy of the CSP global minimum energy structure and V_{CC1} is the molecular volume per CC1.

Structure	Neat			Solvated					
	V_{CC1} (Å^3)	$E_{Latt(Neat)}$ (kJ mol^{-1})	$E_{Latt(Neat)}$ –E_{CSP} (kJ mol^{-1})	V_{CC1} (Å^3)	$E_{Latt(Solvated)}$ (kJ mol^{-1})	$E_{Latt(Solvated)}$ -$E_{Latt(Neat)}$ (kJ mol^{-1})	$E_{Latt(Solvated)}$ -E_{CSP} (kJ mol^{-1})	N	N_{Expt}
CSP_{Min}	1438.8	-183.8	0.0						
α′	1406.2	-177.5	6.3						
β′	1461.3	-177.5	6.2						
Form I (DCM)	1469.6	-163.8	20.0	1481.3	-241.1	-57.3	-77.3	4	4
Form II ($CHCl_3$)	1878.0	-130.0	53.8	1832.9	-234.8	-51.0	-104.8	5	6
Form III (CCl_4)	1969.1	-127.1	56.7	1889.6	-260.9	-77.1	-133.8	5	5
Form IV (EtOAc)	1452.5	-171.2	12.6	1474.7	-219.7	-35.9	-48.5	2	3
Form V (1,4-dioxane)	1581.0	-148.3	35.4	1618.1	-213.2	-29.5	-64.9	3	4
Form VI ($CHCl_3$·oX)	1693.9	-137.4	46.4	1670.4	-218.8	-35.1	-81.4	0	0
Form VII (DCM·oX)	1486.2	-168.2	15.5	1481.7	-199.6	-15.9	-31.4	0	0
Form VIII (pX)	1473.7	-167.6	16.1	1503.7	-197.1	-13.3	-29.4	1	1

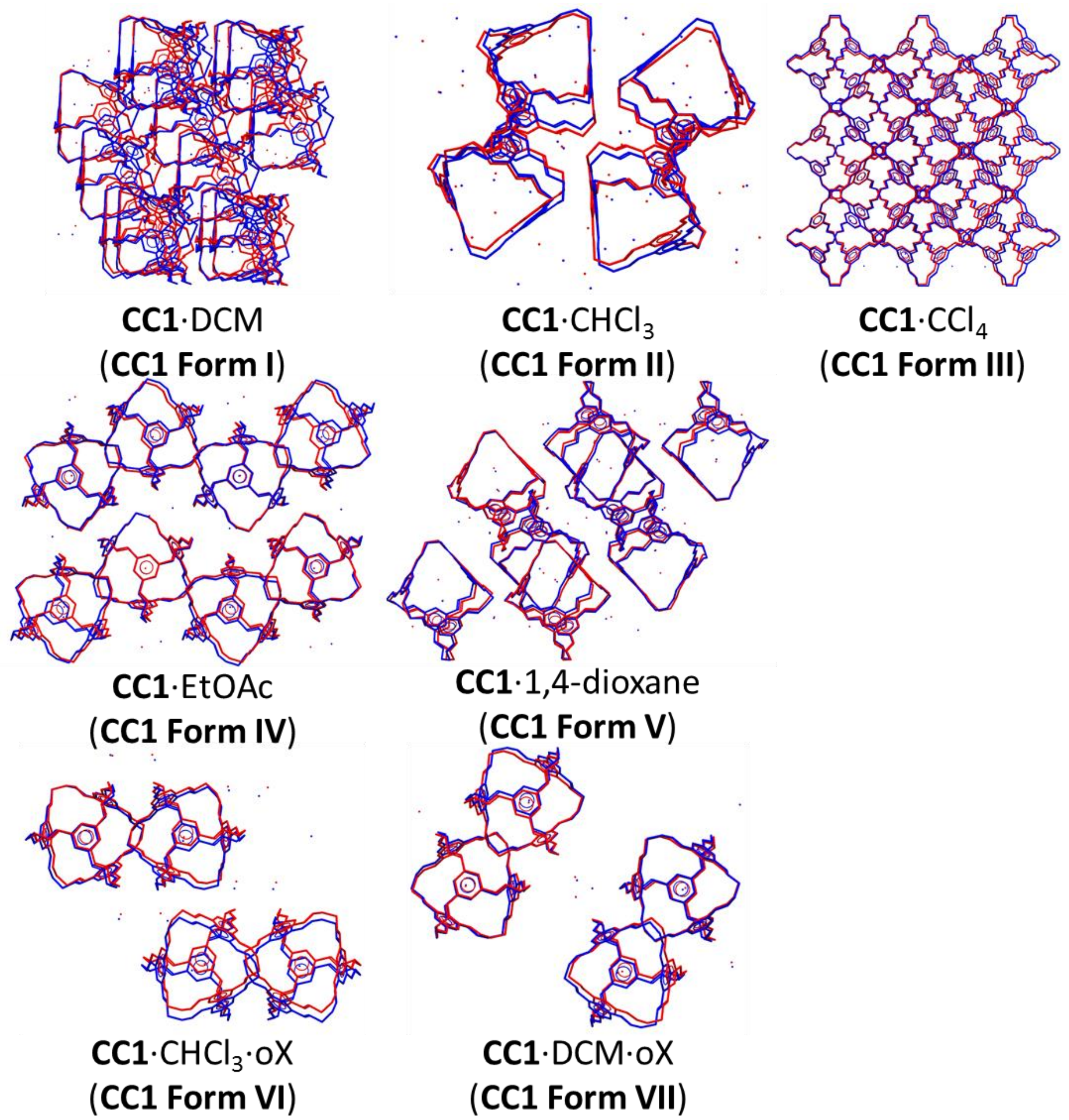

Figure S12. Overlays of the computed (blue) and experimental (red) solvated structures with the solvent represented by a sphere at the solvent centroid position.

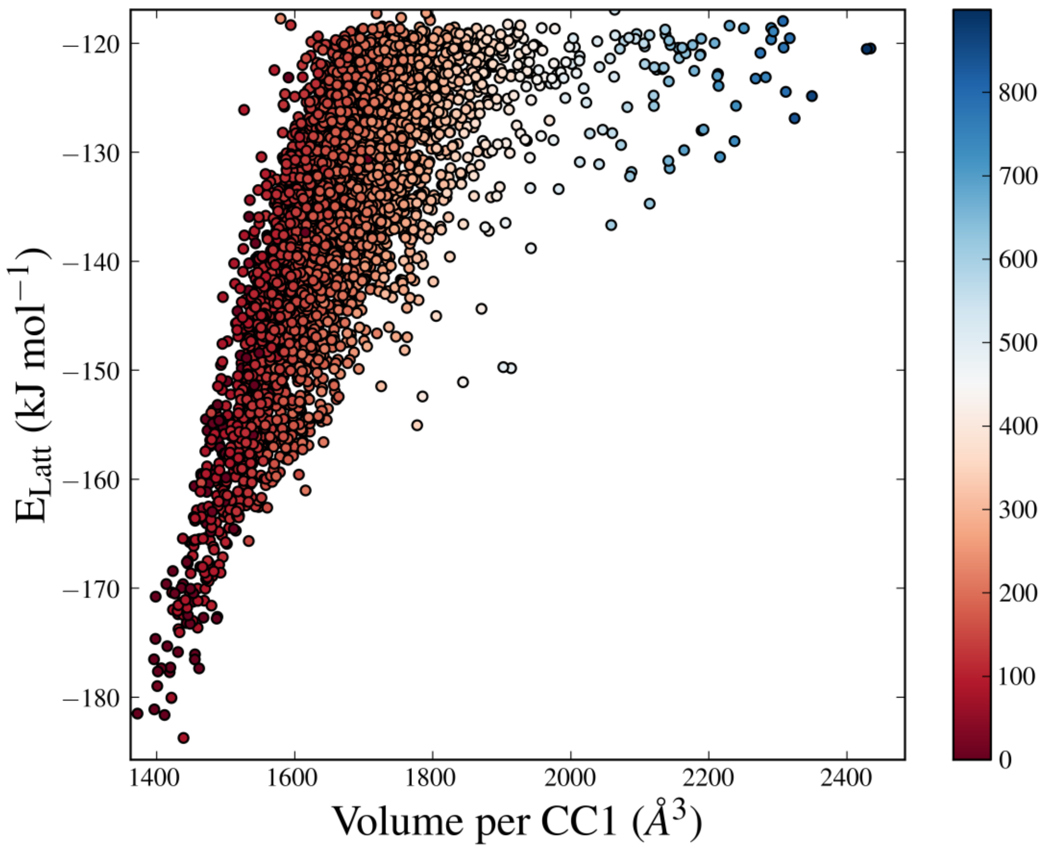

Figure S13. Z'=1 CSP set landscape from Case *et al*.[3] The colour bar gives the accessible volume per **CC1** ($\AA^3$) (as calculated using a 1.2 Å probe).

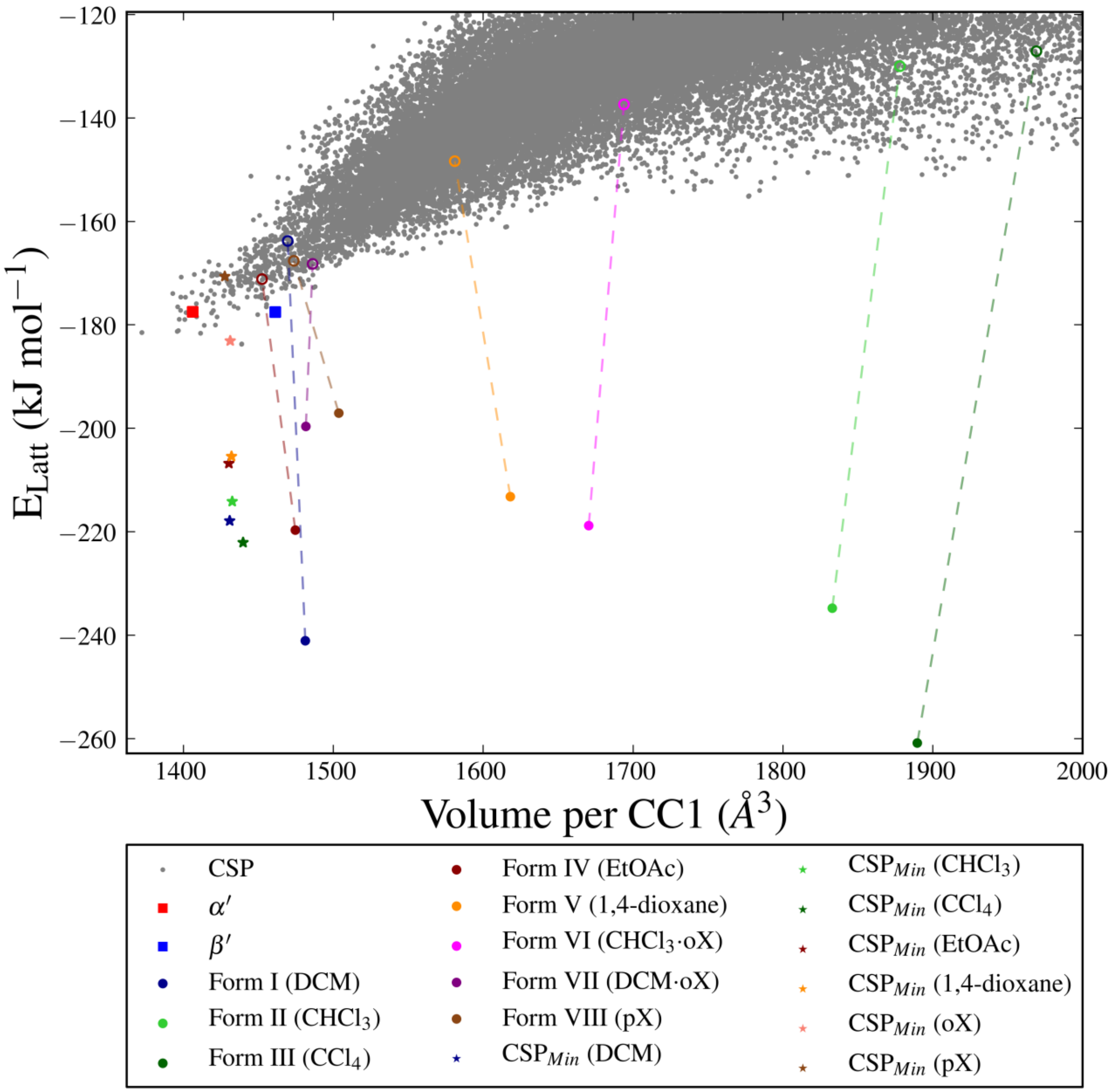

Figure S14. CSP energy landscape for **CC1** (grey points) also showing the calculated latticed energies of the desolvated structures (unfilled circles), after solvent stabilisation (filled circles) and the CSP global minimum structure solvated with the corresponding solvent (stars). Additionally, the experimentally determined α′ and β′ (squares) are given.

Thermal Gravimetric Analysis

Table S10. Comparison of experimentally measured thermal stability (T_{onset} – T_{bp}) and the calculated stabilisation energy (value in brackets from the lattice energy minimised crystal structure).

CC1 + solvent	T_{onset} (°C)	T_{bp} (°C)	$T_{onset} - T_{bp}$ (°C)	Stabilisation energy (kJ mol^{-1})
Form I (DCM)	54	40	14	-77.3
Form II ($CHCl_3$)	83	61	22	-104.8
Form III (CCl_4)	137	77	60	-133.8
Form IV (EtOAc)	76	77	-1	-48.5
Form V (1,4-dioxane)	131	101	30	-64.9 (-93.6)
Form VIII (pX)	141	138	3	-29.4

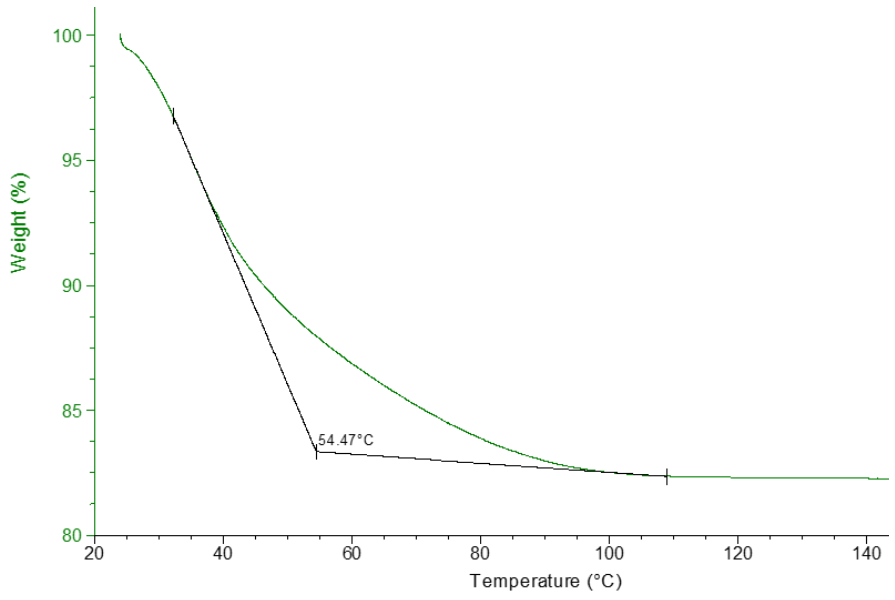

a)

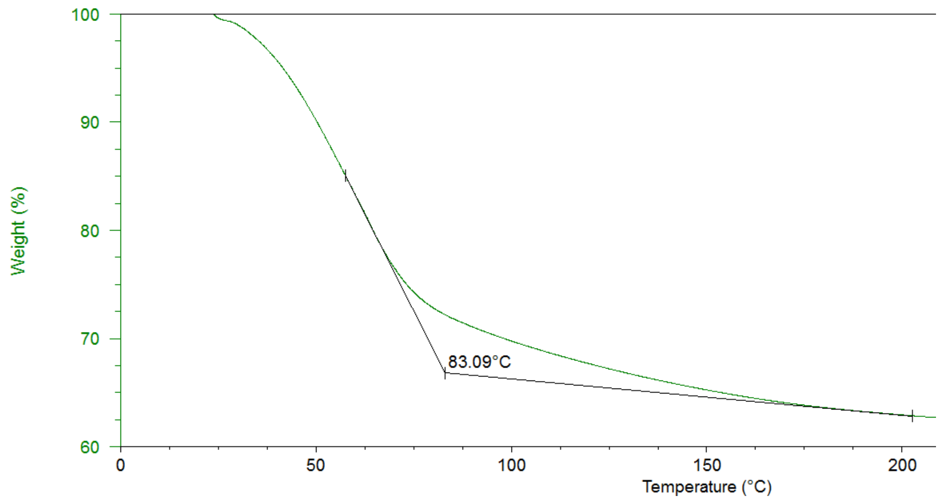

100
90
80
70
60
Weight (%)
83.09°C
0
50
100
150
200
Temperature (°C)

b)

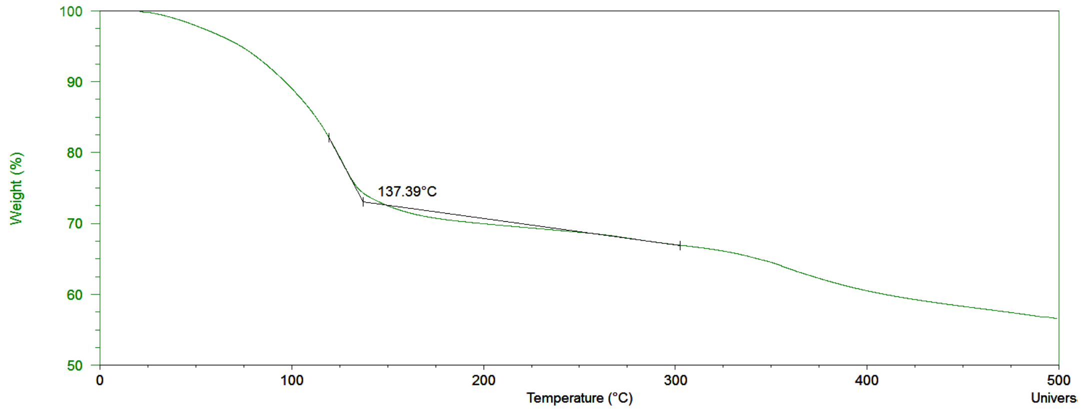

100
90
80
70
60
50
Weight (%)
137.39°C
0
100
200
300
400
500
Temperature (°C)
Univers

c)

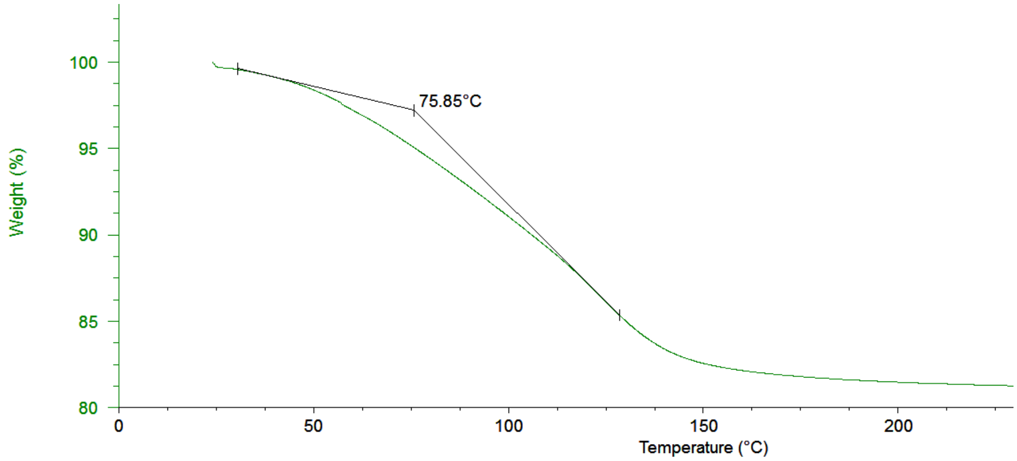

100
95
90
85
80
Weight (%)
75.85°C
0
50
100
150
200
Temperature (°C)

d)

e)

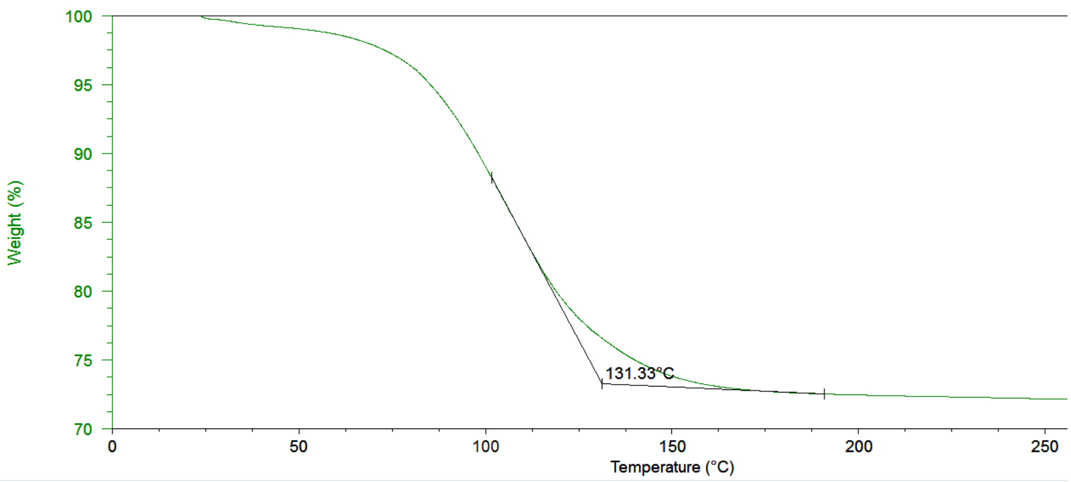

f)

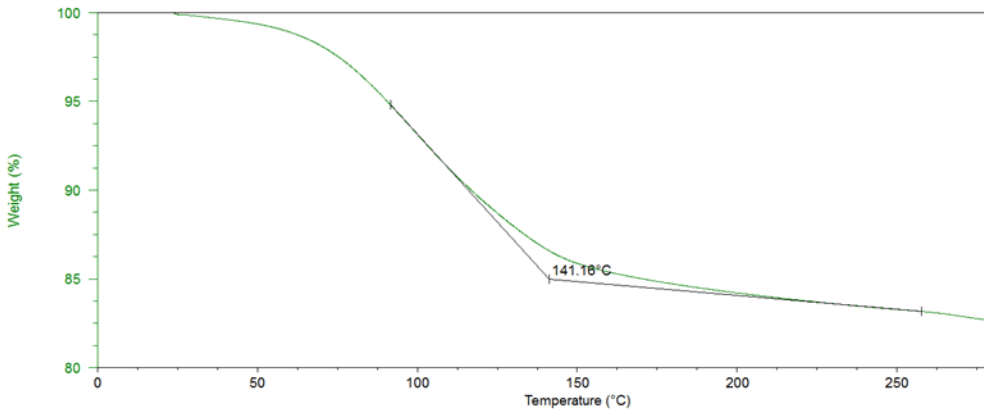

Figure S15. The TGA curve and calculated T_{onset} for a) **Form I,** b) **Form II**, c) **Form III**, d) **Form IV**, e) **Form V**, f) **Form VIII**.

References

1 J. T. A. Jones, D. Holden, T. Mitra, T. Hasell, D. J. Adams, K. E. Jelfs, A. Trewin, D. J. Willock, G. M. Day, J. Bacsa, A. Steiner and A. I. Cooper, *Angew. Chemie Int. Ed.*, 2011, **50**, 749–753.

2 A. Cohelo, TOPAS-Academic (v. 5), Coelho Software, Brisbane, Australia, 2012.

3 D. H. Case, J. E. Campbell, P. J. Bygrave and G. M. Day, *J. Chem. Theory Comput.*, 2016, **12**, 910–924.

4 D. J. F. M. J. Frisch, G. W. Trucks, H. B. Schlegel, G. E. Scuseria, M. A. Robb, J. R. Cheeseman, G. Scalmani, V. Barone, G. A. Petersson, H. Nakatsuji, X. Li, M. Caricato, A. Marenich, J. Bloino, B. G. Janesko, R. Gomperts, B. Mennucci, H. P. Hratchian and J. V. Ort, GAUSSIAN 09 (Revision D.01), Gaussian Inc., Wallingford, CT, 2016.

5 D. E. Williams, *J. Comput. Chem.*, 2001, **22**, 1154–1166.

6 P. J. Winn, G. G. Ferenczy and C. A. Reynolds, *J. Phys. Chem. A*, 1997, **101**, 5437–5445.

7 G. G. Ferenczy, P. J. Winn and C. A. Reynolds, *J. Phys. Chem. A*, 1997, **101**, 5446–5455.

8 R. L. Martin, B. Smit and M. Haranczyk, *J. Chem. Inf. Model.*, 2012, **52**, 308–318.

9 T. F. Willems, C. H. Rycroft, M. Kazi, J. C. Meza and M. Haranczyk, *Microporous Mesoporous Mater.*, 2012, **149**, 134–141.

10 S. L. Price, M. Leslie, G. W. A. Welch, M. Habgood, L. S. Price, P. G. Karamertzanis and G. M. Day, *Phys. Chem. Chem. Phys.*, 2010, **12**, 8478-8490.

11 M. G. Martin, *Mol. Simul.*, 2013, **39**, 1212–1222.

12 A. K. Rappe, C. J. Casewit, K. S. Colwell, W. A. Goddard and W. M. Skiff, *J. Am. Chem. Soc.*, 1992, **114**, 10024–10035.

13 J. A. Chisholm and S. Motherwell, *J. Appl. Crystallogr.*, 2005, **38**, 228–231.
